# Modifiable protective factors for mental health resilience in the offspring of depressed parents: A high‐risk longitudinal cohort spanning adolescence and adulthood

**DOI:** 10.1002/jcv2.12240

**Published:** 2024-05-18

**Authors:** Eglė Padaigaitė‐Gulbinienė, Gemma Hammerton, Victoria Powell, Frances Rice, Stephan Collishaw

**Affiliations:** ^1^ Wolfson Centre for Young People's Mental Health Section of Child and Adolescent Psychiatry Division of Psychological Medicine and Clinical Neurosciences Cardiff University Cardiff UK; ^2^ Centre for Neuropsychiatric Genetics and Genomics School of Medicine Cardiff University Cardiff UK; ^3^ Centre for Academic Mental Health Population Health Sciences Bristol Medical School University of Bristol Bristol UK; ^4^ Medical Research Council Integrative Epidemiology Unit at the University of Bristol Population Health Sciences Bristol Medical School University of Bristol Bristol UK

**Keywords:** depression, longitudinal, mental health, protective, resilience

## Abstract

**Background:**

Several protective factors have been identified for mental health (MH) resilience in adolescent offspring of depressed parents. However, it is unclear if these effects persist into adulthood.

**Methods:**

Depressed parents and their offspring (*N* = 188) from the Early Prediction of Adolescent Depression study were assessed four times (mean offspring ages 12.39, 13.77, 14.82, and 23.41). Mental health resilience was examined using residual scores (better‐than‐expected mood‐, behaviour‐, or anxiety‐related MH at mean age 23 given risk exposure), and categorically as sustained good MH across adolescence and young adulthood.

**Results:**

Only 9.2% of young adults demonstrated sustained good MH. Parents of resilient individuals showed lower comorbidity (anxiety, antisocial behaviour and harmful drinking) and higher depression remission. Considering adolescent protective factors, weak evidence was observed of associations of mood‐resilience with adolescent peer‐relationship quality (*β* = −0.20, 95%CI:−0.36, −0.04); friendship quality (*β* = −0.14, 95%CI:−0.31, 0.02); risk adjustment (*β* = −0.16, 95%CI:‐0.34, 0.03) and dysfunctional attitudes (*β* = 0.18, 95%CI:0.01, 0.35). There was weak evidence of behavioural‐resilience association with parent positive expressed emotion (*β* = −0.15, 95%CI:−0.31, 0.02) and offspring exercise (*β* = −0.37, 95%CI:−0.77, 0.03). No adolescent protective factors showed an association with anxiety‐resilience. For sustained good MH, there was weak evidence of an association with inhibitory control (OR = 0.39, 95%CI:0.14, 1.07). Strong evidence was observed for associations between young adult‐reported peer relationship quality and mood‐resilience (*β* = −0.35, 95%CI:−0.53, −0.17), behavioural‐resilience (*β* = −0.33, 95%CI:−0.51, −0.14) and anxiety‐resilience (*β* = −0.34, 95%CI:−0.53, −0.14), while weak evidence was observed of an association of social activities with anxiety‐resilience (*β* = −0.51, 95%CI:−0.97, −0.06).

**Conclusions:**

We found limited evidence for the long‐lasting effects of adolescent protective factors on adult MH resilience. Social factors remained protective into young adulthood, while family factors did not. Early preventative intervention might not be sufficient to maintain good long‐term MH, and young people will likely require more prolonged support.

**Key points**

- Many factors have been identified as protective for MH resilience in offspring of depressed parents. However, very few studies tracked long‐term protective effects into adulthood.
- We found that resilience is rare while transitioning into adulthood – 9.2% offspring of depressed parents demonstrated sustained good MH.
- Protective factors varied by definition and MH outcome, with peer relationship quality having the most substantial long‐lasting protective effects. Only limited evidence was observed for long‐lasting effect of other adolescent protective factors.
- Our study shows that beneficial effects of adolescent protective factors might not be sufficient to maintain good MH in adulthood. Therefore, more prolonged effort may be required to sustain good MH in offspring of depressed parents.

## INTRODUCTION

About one in five children in the UK lives with a depressed parent (Abel et al., 2019). These children are almost four times more likely to develop depression and other adverse psychiatric outcomes, including anxiety, substance use and conduct disorders (Rice et al., 2002; Thapar et al., 2012; Weissman et al., 2006). Despite the increased familial risk for psychopathology, some do not experience mental health (MH) difficulties or do so only temporarily (Collishaw et al., 2016; Rutter & Quinton, 1984). These individuals could be perceived as resilient – demonstrating relative resistance to psychopathology despite risk exposure (Rutter, 2006; Stainton et al., 2019). One potential explanation for the heterogeneity in MH outcomes could be varying levels of risk exposures. Since parental depression can vary in severity, chronicity and is commonly accompanied by other problems (Downey & Coyne, 1990), resilience in offspring could be explained by exposure to less severe parental depression. Another potential explanation for heterogeneity in offspring MH outcomes is that protective factors buffer children from risks associated with parental depression. However, evidence of protective factors in this high‐risk population is limited since most studies to date have used risk rather than resilience frameworks.

Identifying protective factors is further complicated by the lack of a universally accepted definition of MH resilience (Aburn et al., 2016; Vella & Pai, 2019). Varying conceptual and operational definitions of resilience lead to difficulties in evaluating and comparing findings across studies (Davydov et al., 2010). Nevertheless, most researchers agree on three core components of resilience: the presence of adversity or risk contributing to MH problems, the presence of protective factors that buffer against risk, and better or more positive MH outcomes than would be expected considering the risk (Fletcher & Sarkar, 2013).

Many parent, family, social, cognitive, and lifestyle factors have been identified as protective against poor MH outcomes in offspring of depressed parents, including parental warmth and emotional support, good quality relationships and friendships, self‐efficacy, cognitive and executive functions, positive attitudes, and exercise (Boyd & Waanders, 2013; Brennan et al., 2003; Collishaw et al., 2016; Davidovich et al., 2016; Gunlicks & Weissman, 2008; Mahedy et al., 2018; Ranoyen et al., 2015; Rawal et al., 2013a; Rawal et al., 2014; Riglin et al., 2016). However, most studies have examined protective effects on MH in childhood or adolescence rather than young adulthood – a ‘demographically dense’ period of life characterised by substantial life transitions and a peak age period for the emergence of MH conditions (Caspi et al., 2005; Solmi et al., 2021). Furthermore, it is unclear if these protective effects are general or specific to particular MH outcomes, or if these have long‐lasting effects and could persist into adulthood, since different factors may become relevant as people mature (Ungar & Theron, 2020). Finally, MH resilience is not likely to be monocausal since MH research provides evidence for both multifinality (i.e., the same factors causing different MH outcomes) and equifinality (i.e., different factors can cause the same MH outcomes) (Fried & Robinaugh, 2020). Therefore, a multifactorial approach to MH resilience is needed to examine the long‐term effects of multiple protective factors and their underlying mechanisms (Stainton et al., 2019).

The current study builds on Collishaw and colleagues' (2016) study of MH resilience in adolescent offspring of depressed parents. The authors observed that only a minority of study participants did not develop MH problems during adolescence and that protective effects varied by MH outcome. However, this study had a relatively short follow‐up period (3 years), did not consider cognitive factors, and examined specific factors for mood and behavioural‐resilience but not anxiety. Our study expanded the follow‐up to 13 years and focused on MH resilience into young adulthood. The current study conceptualised MH resilience in two ways. First, MH resilience was operationalised as better‐than‐expected MH outcomes given the degree of parental depression exposure and severity using a residual scores approach (Cahill et al., 2022). The residual scores approach has many strengths, such as capturing variation in risk exposure and an individual's degree of deviation from the ‘norm’, allowing for examination of the role of protective factors for different MH outcomes (Booth et al., 2022; Bowes et al., 2010). However, it does not distinguish between differing longitudinal MH patterns such as resistance (i.e., low MH symptoms over time) or recovery (i.e., ability to bounce back) (Layne et al., 2008; Vella & Pai, 2019). Our work with an advisory group of young adults with lived experience of MH difficulties demonstrated that young adults perceive MH resilience more as a process (i.e., the ability to bounce back) than an outcome (i.e., absence of MH problems) (for more details, see Appendix S1). Hence, a person‐centred approach was also applied to capture MH patterns across multiple outcomes and across time.

Using complementary definitions of MH resilience, we aimed to determine patterns of resilience in young adulthood in this population and examined if:

1. Individuals demonstrating sustained good MH differ in levels of risk exposures, such as parental depression characteristics or social adversity.
2. Previously identified adolescent protective factors have long‐lasting effects on MH resilience into young adulthood, and additionally considered the role of parent depression remission and child cognitive factors.
3. Protective factors vary for mood‐, behavioural‐, and anxiety‐resilience.

## MATERIALS AND METHODS

### Study participants and procedure

The Early Prediction and Adolescent Depression (EPAD) study is a prospective longitudinal high‐risk cohort comprising 337 parents (315 mothers and 22 fathers) with recurrent depression (≥2 DSM‐IV (American Psychiatric Association, 1994) major depressive disorder (MDD) episodes) and their offspring (aged 9–17 years at baseline) predominantly recruited from primary care in South Wales, UK (Mars et al., 2012). If an eligible parent had more than one child, the youngest child was selected to participate in the study. Parents who met the criteria for mania/hypomania and children with moderate to severe intellectual disabilities (IQ < 50) were excluded at study entry.

Over the 13 years of the study period, families were assessed four times at mean offspring age of 12.39 years (range 9–17), 13.77 (range 10–18), 14.82 (range 10–19), and 23.41 (range 18–28). The starting sample for analyses included those who participated in the fourth assessment (*n* = 194). Four participants were excluded for being unexposed to parental depression during their lifetime, while another two were omitted because they had no requisite adult data. The final sample comprised 125–135 participants for complete case analyses, including all relevant data across the four assessments, and 188 participants for imputed data analyses (see Figure S2). In our sample, the main predictors of non‐attendance were family's lower socio‐economic status (i.e., household income, parental education and employment status), lower offspring IQ, and poorer offspring MH (i.e., any DSM disorder and depressive symptoms at baseline) (for details, see Table S3). More details on attrition rates, reasons for non‐participation and other sample characteristics can be found elsewhere (Powell et al., 2021).

### Ethical considerations

Ethical approval for the study was obtained from the Multi‐Centre Research Ethics Committee for Wales and Cardiff University's School of Medicine Ethics Committee. Written informed consent and assent were signed by participants at each wave.

### Measures

**FIGURE 1:**
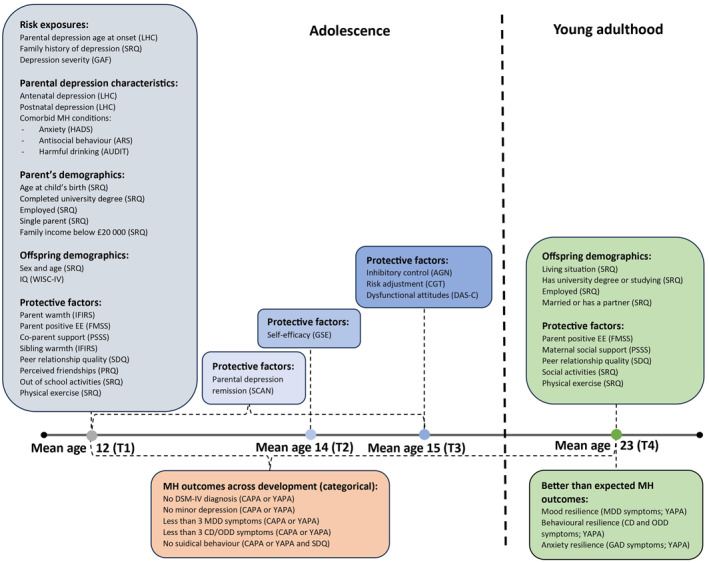
Timing of assessments and measurements used in the study. AGN, Affective Go/No‐Go Task; ARS, Adult Self Report Questionnaire; AUDIT, Alcohol Use Disorders Identification Test; CAPA, The Child and Adolescent Psychiatric Assessment; CD, Conduct disorder; CGT, Cambridge Gambling Task; DAS‐C, The Dysfunctional Attitudes Scale for Children; DSM‐IV, Diagnostic and Statistical Manual of Mental Disorders, fourth edition; FMSS, The Five Minute Speech Sample; GAD, Generalised anxiety disorder; GAF, The Global Assessment of Functioning; GSE, The Generalized Self Efficacy Scale; HADS, The Hospital Anxiety and Depression Scale; IFIRS, The Iowa Family Interaction Rating Scales; LHC, Life History Calendar; MDD, Major depressive disorder; MH, mental health; ODD, Oppositional defiant disorder; PRQ, Peer Relationship Quality Questionnaire; PSSS, Perceived Social Support Scale; SCAN, The Schedule for Clinical Assessment; SDQ, Strengths and Difficulties Questionnaire; SRQ, Self‐reported questionnaire; T1, time point 1; T2, time point 2, T3, time point 3; T4, time point 4; WISC‐IV, The Wechsler Intelligence Scale for Children Fourth Edition; YAPA, the Young Adult Psychiatric Assessment.

All risk exposures, demographic variables and the majority of protective factors were assessed at baseline (mean age 12.39 years), while offspring MH was assessed on four occasions (mean ages 12.39, 13.77, 14.82, and 23.41). The timing of assessments and measurements used in the study are presented in Figure 1, while detailed descriptions on variable types, coding and measurements used are described in Appendix S2.

#### Parental depression

The Schedule for Clinical Assessment (Wing et al., 1990) – a semi‐structured clinical interview was used to obtain information on parental MDD diagnosis and other parental depression characteristics. Two psychiatrists reviewed above‐threshold and sub‐threshold cases, and the final decision on diagnosis was made by clinical consensus.

A number of parental depression characteristics were defined using a life‐history calendar approach to aid retrospective reports (Belli, 1998; Caspi et al., 1996). Parents reported on whether an antenatal and/or postnatal (i.e., up to 1 year after the birth of index offspring) depressive episode occurred with the participating child. Parents also reported any hospitalisations due to depression and gave details of their previous worst two episodes of depression and associated impairment. A severe episode was defined as hospitalisation for depression or an episode of depression with inability to function in all areas (≤30) on the Global Assessment of Functioning Scale (American Psychiatric Association, 1994) as used previously (Collishaw et al., 2016). Information on family history of depression was acquired by asking parents about the number of first and second‐degree relatives (i.e., siblings, parents and grandparents) with depression.

Comorbid parent MH conditions included co‐occurring anxiety, antisocial behaviour and harmful drinking. Anxiety in depressed parents was assessed using the Hospital Anxiety and Depression Scale (Zigmond & Snaith, 1983) anxiety subscale. Parents with a total score of 11 or more – a validated cut‐off score for clinically relevant anxiety – were classified as experiencing anxiety (Sellers et al., 2013; Zigmond & Snaith, 1983). Co‐occurring antisocial behaviour in parents was assessed using 23 items of the Adult Self Report (Achenbach & Rescorla, 2003) questionnaire. A total antisocial behaviour score was dichotomised using a cut‐off of ≥13 (Sellers et al., 2013). The Alcohol Use Disorders Identification Test (Saunders et al., 1993) was used to assess harmful drinking in parents. Alcohol Use Disorders Identification Test is a well‐validated 10‐item questionnaire assessing harmful drinking within the past year. Parents were considered to exhibit harmful drinking if they scored 13 or greater (Saunders et al., 1993; Sellers et al., 2013). Parents scoring above the cut‐off on at least one of these three screening tools were considered as exhibiting a comorbid condition. Parent depression remission prior to the fourth assessment was defined as no depressive episodes at either baseline, second, or third assessment.

#### Parent and young adult demographic characteristics

Information on the index parent's (i.e., age at child's birth, education, employment status, annual family income below £20,000, and being a single parent) and offspring (i.e., age at assessment, sex, and IQ, living situation, education, employment status, and relationship status) demographic characteristics were obtained using a self‐report questionnaire. Education was assessed as the highest qualification obtained and was binary coded as having obtained a university degree or not for parents, or having completed or currently studying for a university degree for offspring. The living situation was determined by asking offspring where they currently live (e.g., parental home, halls of residence, owned or rented accommodation) and by dichotomising this into living with a parent or not (i.e., 1 ‐ yes; 0 – no). Offspring IQ was assessed using the Wechsler Intelligence Scale for Children Fourth Edition (Wechsler, 2003).

#### Mental health resilience

Residual scores (mood‐resilience, behavioural‐resilience and anxiety‐resilience) were generated by regressing MDD symptom counts, oppositional defiant disorder (ODD) plus conduct disorder (CD) symptom counts, and generalized anxiety disorder (GAD) symptom counts onto parental depression characteristics previously shown to be associated with degree of offspring risk for MH difficulties in adolescence (i.e., parent depression age at onset, parent depression severity, and family history of depression) (Collishaw et al., 2016). Residuals from each regression model were saved and used as the outcome measures in further analyses.

A categorical variable was derived, capturing four MH outcome groups: sustained good MH, recovery, adult‐onset disorder, and chronic poor MH. As in the study by Collishaw et al. (2016), individuals who at all assessments (a) did not meet diagnostic criteria for any DSM‐IV (American Psychiatric Association, 1994) diagnosis (i.e., excluding specific phobia), (b) did not meet criteria for minor depression (first three assessments only), (c) experienced less than three symptoms of MDD, (d) experienced less than three behavioural (ODD or CD) symptoms, and (e) did not demonstrate suicide‐related behaviour were considered as meeting criteria for sustained good MH. The recovery group did not meet these criteria for good MH at any of the first three assessments but demonstrated good MH at the fourth assessment. The adult‐onset MH problems group demonstrated good MH over all first three assessments but experienced MH problems as adults. Finally, individuals in the chronically poor MH group did not meet good MH criteria during at least one of the first three assessments and again at the fourth assessment (see Table S1).

Early assessments were made before DSM‐5 (American Psychiatric Association, 2022); therefore, in this study, clinical consensus coding and ratings reflect DSM‐IV (American Psychiatric Association, 1994). Information on adolescents' DSM‐IV (American Psychiatric Association, 1994) diagnoses and symptom counts was obtained using clinical diagnostic interviews at each assessment wave. The Child and Adolescent Psychiatric Assessment (CAPA) was used in the first three assessments, and its adult extension – the Young Adult Psychiatric Assessment (YAPA) was used in the fourth. These are validated interviewer‐based semi‐structured diagnostic interviews to assess the occurrence of psychiatric disorders and symptoms over the preceding 3 months (Angold & Costello, 2000; Angold et al., 1999). The following diagnoses could be made: depressive disorders (i.e., MDD, dysthymia, cyclothymia, bipolar disorder, adjustment disorder, depressive disorder not otherwise specified), anxiety disorders (i.e., GAD, social anxiety, separation anxiety, obsessive‐compulsive disorder, panic disorder, agoraphobia, and specific phobia), eating disorders (i.e., bulimia, eating disorder not otherwise specified), and behavioural disorders (ODD, CD, and disruptive disorder not otherwise specified). Young persons' symptoms were self‐reported and reported by parents at assessments one to three, and were combined by using the highest rating per item at interview‐level. Only young adult‐reported symptoms were used in the fourth assessment. Suicide‐related behaviour was assessed by combining two items (i.e., ‘Suicidal behaviour (attempt)’ and ‘Suicidal thoughts’) from CAPA or YAPA and one item (i.e., ‘I thought about killing myself’) from the Moods and Feelings Questionnaire (Angold & Costello, 1988). Individuals answering affirmatively to at least one of these items were considered as manifesting suicide‐related behaviours.

#### Family, social, cognitive, and lifestyle protective factors

Offspring perceptions of warmth expressed to them by parents and siblings were assessed using The Iowa Family Interaction Rating Scales parental warmth and family interaction rating scales (Collishaw et al., 2016; Melby et al., 1998). An interviewer‐rated five‐minute speech sample of expressed emotion (EE) (Caspi et al., 2004) assessed parent positive EE. Trained researchers coded positive EE according to the tone and content of speech samples (range 0–5), with a higher score indicating more positive EE towards the child (Collishaw et al., 2016). Adolescent‐rated co‐parent emotional support was assessed using the interviewer‐administered Perceived Social Support Scale (Collishaw et al., 2016). Parent‐ and offspring‐reported peer problems from the Strengths and Difficulties Questionnaire (SDQ) assessed adolescent relationship quality with peers (Goodman et al., 2000). Items were reverse coded so a higher score would reflect more positive relationships. Adolescent‐perceived friendship quality was assessed using the Peer Relationship Quality Questionnaire ‐ the 10‐item questionnaire devised for EPAD that evaluates adolescents’ social esteem and peer inclusion and has been shown to have good internal consistency and convergent validity (Collishaw et al., 2016; Rawal et al., 2013b). It also demonstrated adequate reliability and validity in our sample (i.e., Cronbach's *α* = 0.60; correlation with peer relationship quality (i.e., reverse coded SDQ peer problems scale) *r* = 0.57).

Data assessing some cognitive protective factors were unavailable at baseline; therefore, data from the second (self‐efficacy) and third (dysfunctional attitudes, risk adjustment, and inhibitory control) assessments were used. The Generalized Self‐Efficacy Scale (Jerusalem & Schwarzer, 1995) – a well‐validated 10‐item scale assessing an individual's perceived ability to overcome problems and cope with life demands – assessed adolescents' self‐efficacy (Scholz et al., 2002). Dysfunctional attitudes were assessed using The Dysfunctional Attitudes Scale for Children (DAS‐C) (D'Alessandro & Burton, 2006) – a scale comprising 22 items rated on a 6‐point scale that showed sound psychometric properties. The total DAS‐C score was utilised, with a higher score indicating more dysfunctional attitudes (Rawal et al., 2013a; Rice et al., 2015).

Inhibitory control was assessed using the Cambridge Neuropsychological Test Automated Battery (CANTAB) Affective Go/No Go Task (AGN) (Murphy et al., 1999) – a computerised cognitive screening task validated to assess behavioural inhibition (Schulz et al., 2007). Participants were presented with positive and negative words, were given a target valence, and were asked to press a response pad when they saw a word that matched the target valence and withhold a response to non‐target words (distractors). The total number of commission errors (incorrect responses) in all trials was used, and higher errors indicated worse inhibitory control. For reward response, the CANTAB Cambridge Gambling Task – a well‐characterized reward task associated with brain regions involved in the decision‐making process (Clark et al., 2008) was used. During the task, participants received 100 points and tried to maximise their points gained by betting on two possible outcomes (that the target token was in a red or blue box). On each trial, 10 coloured boxes (blue or red) of varying ratios (9:1, 8:2, 7:3, 6:4, 5:5) were presented on the screen in random order. The participant had to choose the box colour and the bet size. This task consisted of 8 blocks of 9 trials in each. The risk adjustment score – how reward‐seeking behaviour is adjusted based on the changing context – was derived as recommended (Clark et al., 2011; Rawal et al., 2013b).

A single parent‐reported item, ‘Attendance at clubs or other organised out‐of‐school activities (at least monthly)’, was used to assess adolescents' engagement in out‐of‐school activities (Collishaw et al., 2016). The self‐reported item ‘Intense exercise or sport more than once a week’ assessed adolescents' physical activity levels (Collishaw et al., 2016).

### Statistical analysis

Analyses were performed using Stata version 17 (StataCorp, 2021). All continuous predictors (see Appendix S2 for more details on variable types) and residuals were standardised to obtain standardised effect size estimates. Univariable and multivariable models were performed for adolescent protective factors (i.e., parental depression remission, parent and sibling warmth, parent positive EE, co‐parent support, peer relationship quality, perceived friendships, out‐of‐school activities, physical exercise, self‐efficacy, inhibitory control, risk adjustment, dysfunctional attitudes). In multivariable models, analyses were adjusted for index parent's education (university degree yes/no), child's biological sex and age.

### Mental health outcomes across development (categorical mental health resilience)

Four categorical MH outcome groups (sustained good MH, recovery, adult‐onset problems, and poor MH) were compared on parental depressive and parent and offspring demographic characteristics. Given the primary aim to test predictors of sustained good MH, logistic regression was employed to test associations between parental depression characteristics, parent and offspring demographic characteristics, and protective factors with sustained good MH (vs all other MH outcome groups combined).

#### Better‐than‐expected mental health outcomes (residual scores)

Linear regression models were utilised to assess the association between resilience residual scores in young adulthood and hypothesised protective factors in adolescence. A negative regression coefficient shows the protective factor is associated with better‐than‐expected MH outcomes, given background risk (for more detailed explanation, see **Figure S3**).

#### Sensitivity analyses

The lack of association between adolescent protective factors and adult outcomes could be due to two reasons: the large gap between assessments or that different protective factors are important for adolescent and adult MH (Jea et al., 2005). To test this, the same protective factors were assessed in early adult life (mean offspring age 23) on measures of MH resilience (using a complete case sample). To rule out the possibility of reverse causation explaining results, we adjusted for offspring's MH problems at baseline (any DSM‐IV diagnosis).

#### Imputed data

To minimise potential bias in the estimates of parameters and to increase sample size, we performed multivariate imputation by chained equations (White et al., 2011) to impute missing data on all variables across all assessments up to our starting sample (those that provided some data at the fourth assessment; *N* = 188). Missing values were imputed using one auxiliary variable for each variable to be imputed – either the same variable assessed at a different time point or a variable related to non‐participation that was also associated with the variable to be imputed. We used 10 cycles of regression‐switching and generated 100 imputed datasets. Convergence plots were used for model diagnostics, and Monte Carlo errors were examined to ensure that 100 imputed datasets were sufficient. Results were combined using Rubin's rules (White et al., 2011). For a detailed description of reasons for non‐participation and imputation procedures, see Appendix S3. Imputation and subsequent analysis code can be found on GitHub: https://github.com/padaigaitee/EPAD‐mental‐health‐resilience.

## RESULTS

Analyses used imputed data. Complete case results were comparable (see Appendix S4).

### Mental health outcomes across development (categorical mental health resilience)

#### Mental health patterns across the study period

Of all young adults who participated, 59.0% were female, and 41.0% were male. Regarding the MH resilience groups, only 9.2% of the sample showed sustained good MH over the 13 years study period, while 18.0% were identified as recovering from MH problems in adulthood. 9.9% developed new‐onset MH problems in young adulthood despite having good MH across adolescence, while the majority (62.9%) exhibited poor MH across the study period.

#### Parental depression and demographics

Table 1 summarises parental depression and parent and young adult characteristics across the MH outcome groups. There was weak evidence for a negative association between parents' comorbid MH conditions and sustained good MH (OR = 0.27, 95%CI:0.06, 1.28). None of the parent or young adult demographic characteristics were associated with sustained good MH except higher adolescent IQ (OR = 1.80, 95%CI:1.03, 3.15).

**TABLE 1:** Parental depression, parent and young adult demographic characteristics according to mental health (MH) outcome group and predictors of sustained good MH (*N* = 188).

		Group				Group comparison
	Total sample (*N* = 188)	Sustained good MH (9.2%)	Recovery (18.0%)	Adult‐onset MH problems (9.9%)	Chronic MH problems (62.9%)	Sustained good MH versus all other groups (reference)
	M (SE) or % (SE)					OR (95%CI)
Parental depression characteristics						
Parental depression age at onset (T1)	26.44 (0.62)	27.95 (2.49)	26.04 (1.69)	31.55 (1.96)	25.52 (0.76)	1.22 (0.70, 2.11)
Number of relatives with a history of depression (T1)	1.50 (0.05)	1.27 (0.11)	1.63 (0.17)	1.34 (0.12)	1.52 (0.06)	0.61 (0.31, 1.22)
Antenatal depression (T1)	12.7% (0.03)	13.0% (0.09)	16.2% (0.07)	‐**◊**	12.7% (0.03)	1.01 (0.21, 4.91)
Postnatal depression (up to 1 year after birth) (T1)	45.2% (0.04)	53.8% (0.13)	46.1% (0.10)	39.5% (0.12)	44.7% (0.05)	1.46 (0.50, 4.25)
Severe episode (GAF <30 or hospitalization) (T1)	29.9% (0.03)	12.2% (0.08)	30.4% (0.09)	‐**◊**	36.4% (0.05)	0.30 (0.06, 1.36)
Comorbid MH conditions (T1)	42.6% (0.04)	19.0% (0.11)	34.2% (0.09)	26.9% (0.11)	50.9% (0.05)	0.27 (0.06, 1.28)
Parent characteristics						
Age at child's birth (T1)	30.01 (0.38)	29.87 (1.44)	30.12 (1.07)	31.93 (1.02)	29.69 (0.50)	0.97 (0.56, 1.70)
Completed university degree (T1)	36.2% (0.04)	40.8% (0.12)	31.3% (0.09)	30.4% (0.11)	37.7% (0.05)	1.24 (0.43, 3.58)
Employed (T1)	75.4% (0.03)	82.6% (0.10)	64.7% (0.09)	81.7% (0.09)	76.3% (0.04)	1.65 (0.39, 6.92)
Single parent (T1)	12.1% (0.02)	10.7% (0.08)	13.3% (0.06)	‐**◊**	13.5% (0.03)	0.80 (0.12, 5.36)
Family income below £20,000 (T1)	25.0% (0.03)	8.6% (0.07)	30.9% (0.09)	6.3% (0.06)	28.7% (0.04)	0.24 (0.03, 1.75)
Young adult characteristics						
Child IQ (T1)	98.39 (0.91)	104.74 (2.43)	95.81 (2.53)	101.43 (2.71)	97.72 (1.16)	1.80 (1.03, 3.15)
Lives with parents (T4)	42.4% (0.04)	31.9% (0.12)	40.6% (0.09)	61.2% (0.14)	41.4% (0.05)	0.61 (0.19, 1.98)
Completed university degree or studying (T4)	59.4% (0.04)	76.9% (0.11)	62.1% (0.09)	‐†	51.9% (0.05)	2.48 (0.69, 8.88)
Employed (T4)	80.3% (0.04)	86.3% (0.09)	84.5% (0.07)	88.1% (0.10)	76.9% (0.05)	1.64 (0.35, 7.69)
Married or lives with a partner (T4)	33.0% (0.04)	37.5% (0.12)	41.9% (0.09)	18.4% (0.11)	32.0% (0.05)	1.24 (0.41, 3.76)

*Note*: MH – mental health; GAF – Global Assessment of Functioning; IQ ‐ intelligence quotient; T1 – time point 1 (mean offspring age 12); T4 – time point 4 (mean offspring age 23); **◊** ‐ parameter estimate could not be estimated due to perfect prediction (nearly 0% prevalence in the group); † **‐** parameter estimate could not be estimated due to perfect prediction (nearly 100% prevalence in the group).

#### Adolescent protective factors

Descriptive statistics for adolescent protective factors across MH outcome groups, plus associations between protective factors and the sustained good MH group (before and after adjusting for confounders) are shown in Table 2. Most parents of young adults exhibiting sustained good MH experienced depression remission over the study period. However, the exact proportion and effect size of this on sustained good MH could not be estimated due to perfect prediction (i.e., within some imputed datasets, 100% of those with sustained good MH had a parent who remitted). Surprisingly, the adult‐onset group scored the highest of all the groups on the majority of protective factors examined, especially on family and lifestyle factors. None of the adolescent protective factors examined were associated with sustained good MH, other than weak evidence for an association between poor inhibitory control and sustained good MH (OR = 0.39, 95%CI:0.14, 1.07).

**TABLE 2:** Protective and risk factors (inhibitory control and dysfunctional attitudes) according to mental health (MH) outcome group and predictors of sustained good MH (*N* = 188).

		Groups				Group comparison	
	Total sample (*N* = 188)	Sustained good MH (9.2%)	Recovery (18.0%)	Adult‐onset MH problems (9.9%)	Chronic MH problems (62.9%)	Sustained good MH versus all other groups (reference)	
	M (SE) or % (SE)					Unadjusted	Adjusted*
						OR (95%CI)	OR (95%CI)
Family factors							
Parent depression remission (T1‐T3)	68.2% (0.03)	‐†	72.6% (0.08)	82.8% (0.09)	60.6% (0.05)	‐†	‐†
Parent warmth (T1)	36.17 (0.46)	34.56 (1.44)	34.67 (1.28)	38.59 (1.31)	36.45 (0.61)	0.78 (0.48, 1.27)	0.75 (0.45, 1.26)
Parent positive EE (T1)	3.41 (0.08)	3.67 (0.22)	3.08 (0.21)	3.80 (0.20)	3.41 (0.10)	1.36 (0.76, 2.43)	1.37 (0.77, 2.46)
Co‐parent support (T1)	2.54 (0.20)	3.40 (0.71)	2.02 (0.49)	4.70 (0.61)	2.22 (0.26)	1.42 (0.83, 2.43)	1.49 (0.85, 2.59)
Sibling warmth (T1)^●^	16.49 (0.39)	15.36 (1.33)	15.83 (0.89)	17.62 (1.35)	16.66 (0.51)	0.95 (0.83, 1.08)	0.93 (0.80, 1.08)
Social factors							
Parent‐reported peer relationship quality (T1)	8.25 (0.14)	8.19 (0.48)	8.22 (0.33)	9.43 (0.24)	8.08 (0.19)	0.97 (0.58, 1.61)	0.98 (0.59, 1.65)
Adolescent‐reported peer relationship quality (T1)	8.13 (0.13)	8.54 (0.34)	8.57 (0.22)	8.26 (0.43)	7.92 (0.18)	1.37 (0.73, 2.59)	1.42 (0.75, 2.71)
Adolescent perceived friendships (T1)	19.76 (0.42)	20.09 (1.46)	21.09 (0.86)	21.68 (0.96)	19.03 (0.57)	1.07 (0.64, 1.79)	1.09 (0.65, 1.82)
Cognitive factors							
Self‐efficacy (T2)	28.27 (0.38)	29.75 (0.90)	27.66 (1.06)	29.67 (0.77)	28.01 (0.50)	1.45 (0.83, 2.51)	1.45 (0.80, 2.62)
Inhibitory control (T3)	15.59 (0.85)	10.12 (2.20)	16.50 (1.98)	11.93 (3.25)	16.71 (1.12)	0.42 (0.16, 1.13)	0.39 (0.14, 1.07)
Risk adjustment (T3)	1.18 (0.06)	1.48 (0.20)	1.05 (0.15)	1.30 (0.21)	1.15 (0.09)	1.45 (0.88, 2.39)	1.46 (0.88, 2.41)
Dysfunctional attitudes (T3)	46.59 (1.18)	41.59 (2.70)	43.29 (2.76)	41.35 (2.84)	49.12 (1.61)	0.66 (0.36, 1.21)	0.64 (0.35, 1.19)
Lifestyle factors							
Out‐of‐school activities (monthly) (T1)	59.6% (0.04)	60.0% (0.13)	57.8% (0.09)	73.2% (0.11)	58.0% (0.05)	1.02 (0.34, 3.09)	1.02 (0.33, 3.20)
Intense physical exercise (> once a week) (T1)	70.9% (0.03)	80.0% (0.10)	62.9% (0.09)	‐†	67.2% (0.05)	1.74 (0.47, 6.39)	1.80 (0.48, 6.73)

*Note*: MH – mental health; EE – expressed emotion; T1 – time point 1 (mean offspring age 12); T2 – time point 2 (mean offspring age 14); T3 – time point 3 (mean offspring age 15); *‐ analyses adjusted for offspring's age, sex, and maternal education (completed university degree) at baseline (T1); † ‐ parameter estimate could not be determined due to perfect prediction (nearly 100% prevalence in the group); ^●^ – sample restricted to those reporting sibling warmth at assessment 1.

### Better‐than‐expected mental health outcomes

#### Parent depression and demographics

None of the parent depression and demographic characteristics were associated with mood or anxiety‐resilience, and only limited evidence of association with behavioural‐resilience was observed (see Table 3) with weak evidence for an adverse effect of parent comorbid MH conditions on behavioural‐resilience (*β* = 0.28, 95%CI:−0.05, 0.61), and strong evidence for university‐degree‐education protective association with behavioural‐resilience (*β* = −0.46, 95%CI:−0.79, −0.14). Contrary to categorical MH outcome analyses, no evidence was observed for an association between IQ and better‐than‐expected MH. There were no associations for other parental depression or parent or young adult demographic factors with MH residual score outcomes.

**TABLE 3:** Associations between parental depression and parent and young adult demographic characteristics with mood‐, behavioural‐, and anxiety‐resilience (residual approach) (*N* = 188).

	Mood‐resilience *β* (95%CI)	Behavioural‐resilience *β* (95%CI)	Anxiety‐resilience *β* (95%CI)
Parental depression characteristics			
Antenatal depression (T1)	−0.25 (−0.73, 0.24)	0.15 (−0.34, 0.63)	0.01 (−0.53, 0.56)
Postnatal depression (T1)	0.11 (−0.23, 0.44)	0.01 (−0.32, 0.33)	0.09 (−0.25, 0.43)
Comorbid MH conditions (T1)	0.18 (−0.15, 0.50)	0.28 (−0.05, 0.61)	0.04 (−0.29, 0.37)
Parent characteristics			
Age at child's birth (T1)	−0.02 (−0.18, 0.15)	−0.01 (−0.18, 0.15)	−0.05 (−0.22, 0.12)
Completed university degree (T1)	−0.27 (−0.60, 0.06)	−0.05 (−0.39, 0.29)	−0.27 (−0.60, 0.07)
Employed (T1)	−0.00 (−0.36, 0.36)	0.00 (−0.36, 0.37)	0.06 (−0.32, 0.43)
Single parent (T1)	−0.31 (−0.83, 0.20)	−0.06 (−0.59, 0.47)	−0.24 (−0.76, 0.27)
Income less than £20,000 (T1)	0.22 (−0.16, 0.61)	0.22 (−0.16, 0.61)	0.13 (−0.26, 0.51)
Young adult characteristics			
Child IQ (T1)	−0.05 (−0.22, 0.11)	−0.04 (−0.21, 0.13)	0.06 (−0.10, 0.23)
Lives with parents (T4)	0.00 (−0.34, 0.34)	0.13 (−0.20, 0.47)	−0.27 (−0.60, 0.07)
Has a university degree or studying (T4)	−0.26 (−0.59, 0.08)	−0.46 (−0.79, −0.14)	−0.10 (−0.44, 0.24)
Employed (T4)	−0.32 (−0.76, 0.11)	−0.06 (−0.47, 0.36)	−0.27 (−0.72, 0.18)
Married or has a partner (T4)	−0.16 (−0.50, 0.19)	−0.11 (−0.44, 0.23)	0.07 (−0.27, 0.42)

*Note*: IQ ‐ intelligence quotient; T1 – time point 1 (mean offspring age 12); T3 – time point 3 (mean offspring age 15); T4 – time point 4 (mean offspring age 23).

#### Adolescent protective factors

Results of analyses examining adolescent protective factors as predictors of mood‐, behavioural‐, and anxiety‐resilience are presented in Table 4. Of all hypothesised protective factors, only social and cognitive factors were associated with mood‐resilience. Weak evidence was observed for association of adolescent‐reported peer relationship quality (*β* = −0.20, 95%CI:−0.36, −0.04) and friendships (*β* = −0.14, 95%CI:−0.31, 0.02) with mood‐resilience. We also observed weak evidence for a protective effect of risk adjustment (*β* = −0.16, 95%CI:−0.34, 0.03) and a risk effect of dysfunctional attitudes (*β* = 0.18, 95%CI:0.01, 0.35) on mood‐resilience. For behavioural‐resilience, weak evidence was observed for protective effects of parent positive EE (*β* = −0.15, 95%CI:−0.31, 0.02) and exercise (*β* = −0.37, 95%CI:−0.77, 0.030). None of the hypothesised adolescent protective factors were associated with anxiety‐resilience.

**TABLE 4:** Adolescent protective and risk factors (dysfunctional attitudes and inhibitory control) association with better‐than‐expected mental health (MH) outcomes in young adulthood (*N* = 188).

	Mood‐resilience		Behavioural‐resilience		Anxiety‐resilience	
	Unadjusted *β* (95%CI)	Adjusted* *β* (95%CI)	Unadjusted *β* (95%CI)	Adjusted* *β* (95%CI)	Unadjusted *β* (95%CI)	Adjusted* *β* (95%CI)
Family factors						
Parent depression remission (T1‐T3)	−0.16 (−0.51, 0.19)	−0.18 (−0.53, 0.17)	−0.19 (−0.54, 0.15)	−0.25 (−0.59, 0.09)	−0.24 (−0.60, 0.12)	−0.24 (−0.59, 0.12)
Parent warmth (T1)	0.13 (−0.04, 0.29)	0.14 (−0.04, 0.31)	0.01 (−0.16, 0.18)	−0.05 (−0.23, 0.13)	0.10 (−0.08, 0.28)	0.13 (−0.05, 0.32)
Parent‐positive EE (T1)	0.01 (−0.15, 0.18)	0.01 (−0.16, 0.17)	−0.12 (−0.29, 0.05)	−0.15 (−0.31, 0.02)	−0.06 (−0.24, 0.11)	−0.06 (−0.23, 0.12)
Co‐parent support (T1)	0.07 (−0.09, 0.24)	0.05 (−0.12, 0.22)	0.10 (−0.07, 0.26)	0.06 (−0.12, 0.23)	−0.02 (−0.18, 0.15)	−0.02 (−0.19, 0.15)
Sibling warmth (T1)^●^	0.07 (−0.12, 0.25)	0.06 (−0.13, 0.26)	−0.11 (−0.27, 0.05)	−0.09 (−0.26, 0.08)	0.11 (−0.08, 0.29)	0.07 (−0.13, 0.26)
Social factors						
Parent‐reported peer relationship quality (T1)	−0.05 (−0.21, 0.11)	−0.06 (−0.23, 0.10)	−0.03 (−0.20, 0.14)	−0.03 (−0.20, 0.14)	0.08 (−0.08, 0.24)	0.06 (−0.10, 0.22)
Adolescent‐reported peer relationship quality (T1)	−0.17 (−0.33, −0.02)	−0.20 (−0.36, −0.04)	−0.14 (−0.34, 0.05)	−0.13 (−0.33, 0.06)	−0.07 (−0.22, 0.09)	−0.11 (−0.27, 0.05)
Adolescent perceived friendships (T1)	−0.13 (−0.29, 0.03)	−0.14 (−0.31, 0.02)	−0.14 (−0.32, 0.05)	−0.14 (−0.32, 0.04)	−0.03 (−0.20, 0.14)	−0.05 (−0.22, 0.12)
Cognitive factors						
Self‐efficacy (T2)	−0.04 (−0.21, 0.14)	0.01 (−0.18, 0.19)	−0.05 (−0.25, 0.14)	−0.02 (−0.22, 0.18)	−0.01 (−0.18, 0.17)	0.02 (−0.16, 0.21)
Inhibitory control (T3)	0.04 (−0.14, 0.22)	0.01 (−0.19, 0.20)	0.02 (−0.20, 0.25)	−0.05 (−0.29, 0.18)	−0.13 (−0.30, 0.04)	−0.13 (−0.31, 0.05)
Risk adjustment (T3)	−0.16 (−0.34, 0.02)	−0.16 (−0.34, 0.03)	−0.08 (−0.27, 0.11)	−0.08 (−0.27, 0.10)	−0.03 (−0.20, 0.14)	−0.02 (−0.20, 0.15)
Dysfunctional attitudes (T3)	0.16 (−0.01, 0.33)	0.18 (0.01, 0.35)	0.11 (−0.08, 0.30)	0.13 (−0.06, 0.31)	0.10 (−0.06, 0.27)	0.11 (−0.05, 0.27)
Lifestyle factors						
Out‐of‐school activities (monthly) (T1)	0.11 (−0.23, 0.46)	0.13 (−0.23, 0.48)	0.03 (−0.34, 0.40)	−0.01 (−0.39, 0.36)	0.01 (−0.33, 0.34)	0.02 (−0.32, 0.36)
Intense physical exercise (> once a week) (T1)	−0.14 (−0.51, 0.22)	−0.17 (−0.54, 0.20)	−0.26 (−0.66, 0.14)	−0.37 (−0.77, 0.030	−0.26 (−0.62, 0.11)	−0.23 (−0.60, 0.15)

*Note*: Negative regression coefficient shows positive association between protective factor and outcome; MH – mental health; EE – expressed emotion; T1 – time point 1 (mean offspring age 12); T2 – time point 2 (mean offspring age 14); T3 – time point 3 (mean offspring age 15); *‐ analyses adjusted for offspring's age, sex, and maternal education (completed university degree) at baseline (T1); ^●^ – sample restricted to those reporting sibling warmth at assessment 1.

### Sensitivity analyses

#### Mental health outcomes across development (categorical mental health resilience) and young adult protective factors

The adult‐onset MH outcome group again scored relatively highly across different young adult protective factor measures (see Table S7). None of the young adulthood factors examined were associated with sustained good MH. Results did not change after adjustment of baseline confounders.

#### Better‐than‐expected mental health and young adult protective factors

Strong evidence was observed for associations between self‐ but not parent‐reported adult peer relationship quality with mood‐ (*β* = −0.35, 95%CI:−0.53, −0.17), behavioural‐ (*β* = −0.33, 95%CI:−0.51, −0.14), and anxiety‐resilience (*β* = −0.34, 95%CI:−0.53, −0.14), with weaker evidence for social activities in young adulthood and anxiety‐resilience (*β* = −0.51, 95%CI:−0.97, −0.06), after adjusting for confounders and offspring MH disorders at baseline (see Table S8).

## DISCUSSION

The current study aimed to estimate the prevalence of MH resilience in adult offspring of depressed parents, test if resilient individuals experience differing levels of risk exposures and examine if previously established adolescent protective factors have a long‐lasting, general, or specific effect on resilient outcomes in young adulthood. Using a high‐risk cohort spanning over a decade, we found that among offspring of depressed parents, resilience was rare ‐ only one in 10 did not develop MH problems, while one in 5 demonstrated improvement in MH during young adulthood. This confirms very high rates of MH problems in the offspring of depressed parents (Collishaw et al., 2016; Feder et al., 2009), and that young adulthood is a high‐risk period for developing MH problems (Solmi et al., 2021).

Resilient and non‐resilient young adults demonstrated relatively similar levels of risk exposures, indicating that MH resilience is not only a result of varying levels of risk exposures in line with the findings of Loechner et al. (2020). Furthermore, parental depression remission was identified as protective for offspring's MH outcomes. This implies that parental depression could be a modifiable protective factor and is consistent with studies demonstrating that parental depression remission may improve offspring's MH outcomes (Cuijpers et al., 2015; Gunlicks & Weissman, 2008).

We observed limited evidence for the long‐lasting effects of previously identified adolescent protective factors for MH resilience in young adulthood. Previous studies demonstrated family, social, cognitive, and lifestyle associations with MH resilience in adolescence (Collishaw et al., 2016; Davidovich et al., 2016; Mahedy et al., 2018). However, of all protective factors examined, we only observed weak evidence for family (i.e., positive EE), cognitive (i.e., inhibitory control, high risk adjustment, and dysfunctional attitudes) and lifestyle (i.e., exercise) factors. Engagement in social activities assessed in adulthood but not adolescence was associated with anxiety‐resilience. Considering its multifaceted nature, it would be important to disentangle if these protective effects are driven by the characteristics of a young person (i.e., being proactive), family factors such as affordability, or community factors such as access to and provision of these activities in schools and neighbourhoods. Peer relationship quality had the most substantial long‐lasting protective effects, which were observed across different MH outcomes and irrespective of the timing of assessment (adolescence or young adulthood). This is consistent with evidence that social relationships and social support are crucial for preventing and recovering from MH problems (Bjørlykhaug et al., 2022). Further analyses revealed that the lack of long‐lasting protective effects for other previously identified factors was not due to the significant time gap between protective factors and MH outcomes in this study (i.e., as adult measures of the same factors were also not associated with adult resilience). Instead, it suggests there may be shifting priorities while transitioning into adulthood, indicating that protective factors could be developmental‐stage specific. In particular, findings from this and previous papers (Collishaw et al., 2016; Mahedy et al., 2018) suggest that the effect of direct parental and family influences might lessen while cognitive, social, and lifestyle influences become stronger while transitioning into adulthood. This is in line with longitudinal genetic epidemiology studies demonstrating that unlike shared environment that is stable and long‐term, non‐shared environmental factors are often developmental‐stage specific and often do not seem to persist over time (Burt et al., 2015; Hopwood et al., 2011; Zheng et al., 2016).

Interestingly, protective effects varied by definition and MH outcomes. Most protective factors identified were associated with mood but not behaviour or anxiety‐resilience. Furthermore, factors having protective effects for better‐than‐expected MH outcomes did not predict sustained good MH and vice versa. As Nishimi et al. (2021) found, our results demonstrate that the protective factors identified depend on the definition of MH resilience.

Finally, offspring who developed MH problems as adults had comparable levels of risk exposures and protective effects as those who sustained good MH over the study period and did not develop MH problems. These unexpected results might have a theoretical explanation but might also be explained by collider bias. By conditioning on a collider (e.g., restricting to those with no MH problems at baseline when directly comparing adult‐onset with sustained good MH), we could have induced alternative/indirect paths between protective factors (e.g., perceived friendship) and outcome (e.g., adult‐onset and sustained good MH comparison), thus observing group differences that result from collider bias rather than actual group differences (see Figure S4 for a detailed explanation). Alternatively, considering that both groups of individuals were identified as resilient during adolescence in previous reports on this sample (Collishaw et al., 2016), it could be postulated that adolescent protective factors may delay rather than prevent MH problems. Thus, MH resilience may change over development particularly around key risk/transition periods (Solmi et al., 2021). Alternatively, MH problems observed in the adult‐onset group could be short‐term, resulting from stressful experiences or life challenges while transitioning into adulthood that were not assessed. Indeed, young adulthood is considered a ‘demographically dense’ period of life, and prospective studies demonstrate that MH problems in early adulthood might be relatively normative (Caspi et al., 2005; Moffitt et al., 2010; Solmi et al., 2021).

### Strengths and limitations

The current study has important strengths, including using one of the largest high‐risk studies that followed parents with depression and their offspring for over a decade, a prospective longitudinal study design, and a range of MH conditions examined using semi‐structured clinical diagnostic interviews with multiple informants. Different approaches were used to operationalise MH resilience and analyses were adjusted for well‐established confounders – offspring sex, age, and maternal education. Additionally, by using a residual scores approach, our analyses also accounted for alternative explanations for MH resilience in offspring of depressed parents – differences in the severity of parental depression – that is rarely done in resilience research. Nevertheless, like all observational studies, our study is still susceptible to bias from unmeasured confounding, and future studies could consider the inclusion of other potential confounders such as poverty, stress or life events. Although multiple imputation was used to increase the available sample size by including participants with partially complete data and to account for potential attrition bias in estimates, we only imputed up to the starting sample of those who provided some data at the fourth assessment (*N* = 188; 56% of original sample). Therefore, there may still be some bias in the exposure‐outcome associations. Our study could also be affected by low statistical power increasing the likelihood of false negatives and affecting the precision of our estimates – resulting in wider confidence intervals, while multiple testing could also lead to false positives (Button et al., 2013). Particular caution is needed in comparisons of small subgroups, such as those with sustained good MH. Furthermore, participating parents were recruited for this study on the basis of recurrent major depressive episodes, mostly from primary care in Wales. In the UK, over 95% of adult depression cases are treated in primary care (Ramanuj et al., 2019), and thus, the sample likely represents well this population of depressed parents. Nevertheless, our study might not capture parents who did not seek treatment and results might not be generalisable to less severe parental depression cases. Finally, our sample was predominantly maternal, potentially due to higher lifetime depression prevalence and preponderance to recurrent and chronic depression in females compared to males and males being less likely to seek help from medical doctors or participate in health studies (Markanday et al., 2013; Piccinelli & Wilkinson, 2000; Susukida et al., 2015). Thus, our results might not be fully generalisable to the offspring of depressed fathers. Future studies could examine the role of paternal MH on offspring MH outcomes.

## CONCLUSION

Our study confirms high rates of MH problems and limited evidence for the long‐lasting effects of adolescent protective factors, with peer relationship quality being most strongly supported. Protective effects observed were developmental‐stage specific and varied by the definition of MH resilience and MH outcome. This implies that contrary to common perception, exposure to early protective effects might not be sufficient to maintain good MH in young adulthood, and more prolonged effort may be required to support young people with a depressed parent. Rather than simply focussing on the prevention of MH problems in this population, future longitudinal studies with larger sample sizes could prioritise the identification of protective factors that could help to delay or recover from MH problems.

## AUTHOR CONTRIBUTIONS

**Eglė Padaigaitė‐Gulbinienė**: Conceptualization; Formal analysis; Investigation; Methodology; Project administration; Writing – original draft; Writing – review & editing. **Gemma Hammerton**: Conceptualization; Formal analysis; Investigation; Methodology; Supervision; Writing – original draft; Writing – review & editing. **Victoria Powell**: Conceptualization; Formal analysis; Investigation; Methodology; Project administration; Writing – original draft; Writing – review & editing. **Frances Rice**: Conceptualization; Data curation; Formal analysis; Funding acquisition; Investigation; Methodology; Project administration; Supervision; Writing – original draft; Writing – review & editing. **Stephan Collishaw**: Conceptualization; Data curation; Formal analysis; Funding acquisition; Investigation; Methodology; Project administration; Supervision; Writing – original draft; Writing – review & editing.

## CONFLICT OF INTEREST STATEMENT

The authors have declared that they have no competing or potential conflicts of interest.

## ETHICAL CONSIDERATIONS

Ethical approval was granted by the Multi‐Centre Research Ethics Committee for Wales and from the School of Medicine Ethics Committee, Cardiff University.

## Supporting information

Supplementary Material

## Data Availability

The data that support the findings of this study are available from the corresponding author upon reasonable request.
