# Supplementary material for: Modifiable protective factors for mental health resilience in the offspring of depressed parents: A high‐risk longitudinal cohort spanning adolescence and adulthood
Source: JCPP Adv. 2024 May 18;4(3):e12240. doi: 10.1002/jcv2.12240 (PMC11472801; doi:10.1002/jcv2.12240)
Supplement: Supplementary file 1 — Supplementary Material [file JCV2-4-e12240-s001.docx]

**Supporting Information**

**Appendix S1. Adult offspring of depressed parents advisory group results**

***Figure S1.*** *Youth advisory group ranking of the key protective factors categories for mental health resilience in order of importance*

**Figure S2. Participation and attrition rates over the study period**

**Table S1. Classification of young adults in four different mental health categories**

**Figure S3. Residual approach**

**Appendix S2. Description of measures and variables used in the study**

**Appendix S3. Missing data considerations and multiple imputation**

***Table S2.*** *Characteristics of imputed variables*

***Table S3.*** *Reasons for non-participation*

**Appendix S4. Complete cases results – risk exposures and protective factors assessed in adolescence**

***Table S4.*** *Risk exposures and categorical mental health outcomes*

***Table S5.*** *Risk exposures and better-than-expected mental health outcomes*

***Table S6.*** *Protective factors and categorical mental health outcomes*

***Table S7.*** *Protective factors and better-than-expected mental health outcomes*

**Appendix S5. Complete cases results – protective factors assessed in young adulthood**

***Table S8.*** *Protective factors and categorical mental health outcomes*

***Table S9.*** *Protective factors and better-than-expected mental health outcomes*

**Figure S4. Collider bias**

**References**

**Appendix S1. Adult offspring of depressed parents advisory group results**

To identify the best approach to define mental health resilience and determine the protective factors that should be prioritised, we consulted an advisory group with lived experiences of mental health difficulties. We undertook the focus group via videoconferencing using the Zoom platform with 5 adult offspring (4 females and 1 male; 24-30 years old) of depressed parents recruited from the same sample – Early Prediction of Adolescent Depression (EPAD) study. Most of them were from Wales, worked or studied full-time and experienced mental health problems in the past.

Considering the definition of mental health resilience, young adults agreed that it could mean different things to different people: “It can mean two things - living with things when it gets bad or not letting those things get to that point “. Nevertheless, they did not like the outcome definition of mental health resilience (i.e., not developing mental health problems despite the adversity) since it implies that “you cannot be resilient if you had mental health problems in the past“ and prefered to define resilience as a process (i.e., ability to bounce back from adversities). Young adults believed that resilience could be perceived as a “general preparedness from everything that comes your way “ or “emotional memory “ and can be built and maintained by learning from adverse experiences: “Your body remembers that you went through this already and helps to be more resilient the second time something similar happens “. They also pointed out that it is not constant – “You can build resilience, but you can also witter it away “.

When asked about protective factors for mental health resilience, young adults agreed that “resilience starts from within “ and put the emphasis on cognitive factors such as self-efficacy, coping skills, emotional strength (i.e., regulation), and being proactive (see **Figure S1**). The second most crucial domain was lifestyle factors such as self-care, good night’s sleep, exercise, hobbies (“having a life outside of the family”), and ability to “distract/ remove yourself from the situation “. Emotional support – “knowing people who can listen rather than give advice” and a good support network were also considered necessary since it can be “something constant while going through changes”. Community and society-level protective factors, such as reducing mental health-related stigma and improving mental health education, were considered less important, while biological factors were considered least important. Young adults believed that resilience is “more about your environment and personality than about biology”, although they agreed that genetics could also play a role.

To sum up, adult offspring of depressed parents believed that mental health resilience could be built and maintained by learning from adverse experiences and preferred to define resilience as a process rather than an outcome. They identified cognitive and lifestyle factors as the most relevant ones to focus on, demonstrating a proactive approach and eagerness to improve their mental health and build a better future without passively waiting for external support.


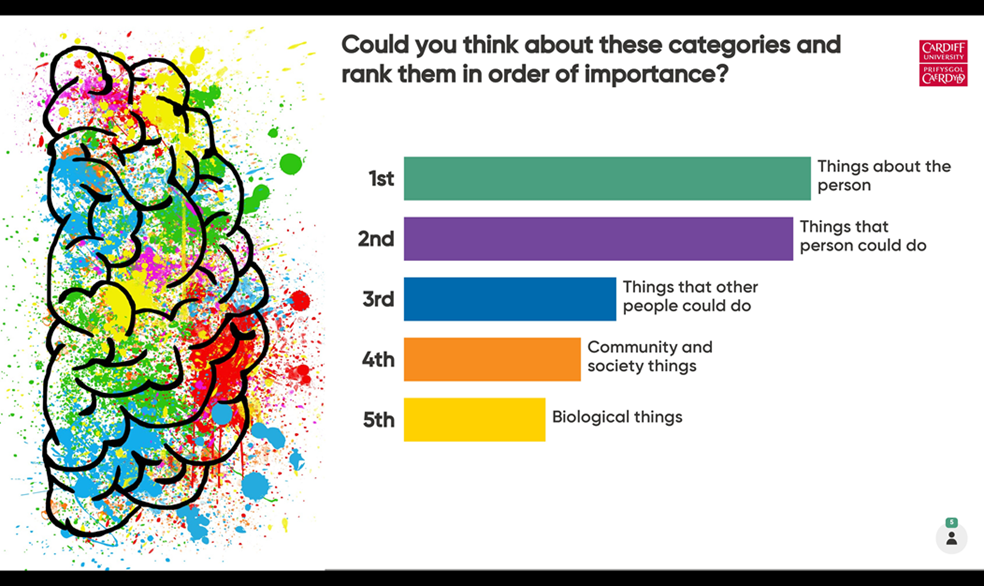


***Figure S1.*** *Youth advisory group ranking of the key protective factors categories for mental health resilience in order of importance. The image was obtained using the interactive presentation software Mentimeter.*

**Figure S2. Participation and attrition rates over the study period**


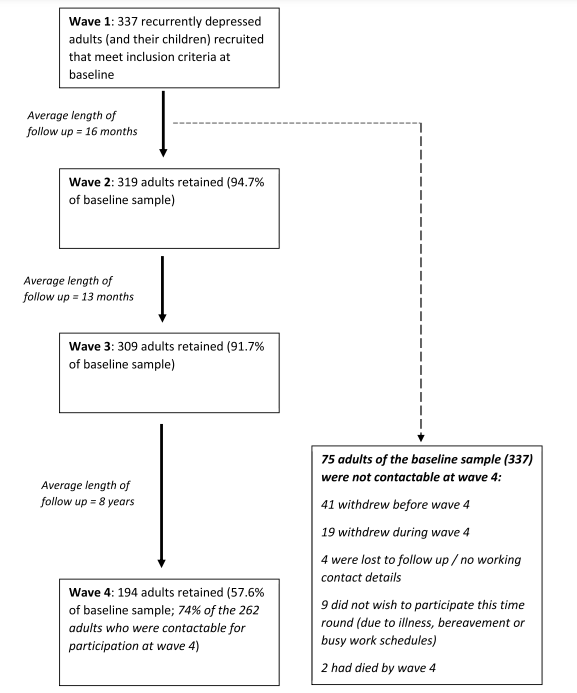


***6 adults of the wave 4 sample (194) were excluded from imputed data analyses:***

*4 were unexposed to parental depression during their lifetime*

*2 had no requisite adult data*

**Wave 4 imputed data sample**: comprised of 188 adults (55.8% of baseline sample; 96.9% of those who participated at wave 4).

This figure was adapted from Powell et al. (2021) and updated to include exclusion criteria and imputed data sample sections at assessment 4.

**Table S1. Classification of young adults in four different mental health categories based on their mental health outcome at each assessment**

| **Developmental period** | **Good MH criteria met at each assessment** | **Sustained good MH** | **Recovery** | **Adult-onset problems** | **Chronic poor MH** |
| --- | --- | --- | --- | --- | --- |
| **Adolescence** | T1 | **✓** | ✘ | **✓** | ✘ |
|  |  | AND | OR | AND | OR |
|  | T2 | **✓** | ✘ | **✓** | ✘ |
|  |  | AND | OR | AND | OR |
|  | T3 | **✓** | ✘ | **✓** | ✘ |
|  |  | AND | AND | AND | AND |
| **Young adulthood** | T4 | **✓** | **✓** | ✘ | ✘ |

**Figure S3. Residual approach**


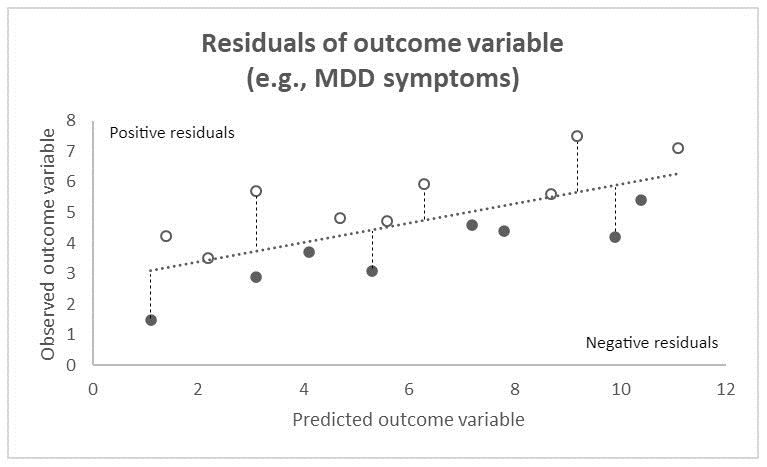


This figure shows hypothetical positive and negative residuals obtained from regression analysis by regressing major depressive disorder (MDD) symptoms on parental depression severity, age of onset and family history of depression. Positive residuals (unfilled circles) reflect values greater than expected considering the risk exposure – in this example, more MDD symptoms than expected, considering parental depression characteristics. Negative residuals (filled circles) show individuals scoring lower than expected on depressive symptoms considering the level of risk exposures. Therefore, in analyses considering protective factor associations with residual score, a negative regression coefficient indicates that protective factor is associated with a better-than-expected mental health outcome.

**Appendix S2. Description of measures and variables used in the study**

| **Measure** | | **Construct** | | | | |
| --- | --- | --- | --- | --- | --- | --- |
| **Name** | **Description** | **Name** | **Coding** | **How reported** | **Type of variable** | **Time of assessment and (offspring age)** |
| **Parental depression and demographic characteristics** | | | | | | |
| **Life History Calendar (LHC) approach**  (Caspi et al., 1996) | A method that combines a visual calendar with a semi-structured interview to map out life events and their onset, duration, sequencing, and co-occurrence. This method provides visual cues for the interviewer and study participant and aids the retrospective recall | Parental depression age at onset | Age when a parent was first diagnosed with MDD. A higher number indicates later depression onset | Retrospectively | Continuous | T1 (mean age 12) |
|  |  | Family history of depression | Number of depressed individuals among first- and second-degree relatives (i.e., siblings, parents, grandparents) of a child (parent-reported). Higher score indicates more relatives with a history of MDD | Retrospectively | Continuous | T1 (mean age 12) |
|  |  | Antenatal depression | The mother was diagnosed with MDD during pregnancy | Retrospectively | Binary  (1 – yes, 0 – no) | T1 (mean age 12) |
|  |  | Postnatal depression | The mother was diagnosed with MDD within the first year of birth | Retrospectively | Binary  (1 – yes, 0 – no) | T1 (mean age 12) |
| **The Schedule for Clinical Assessment (SCAN)**  (Wing et al., 1990) | A semi-structured interview is used to diagnose DSM-IV disorders based on the symptoms and impairment reported in the last month. A trained clinician administered it | Parental depression remission | Parents were not meeting MDD diagnosis at either baseline, second, or third assessment. Information on MDD diagnosis from all three waves was combined and dichotomised into not meeting MDD diagnosis in at least one assessment wave. It did not consider retrospectively reported depressive episodes between assessments | Prospectively | Binary  (1 – yes, 0 – no) | T1 (mean age 12), T2 (mean age 14)  T3 (mean age 15) |
| **The Global Assessment of Functioning (GAF)**  (American Psychiatric Association, 1994) | A screening tool that measures how individuals' mental health symptoms affect their functioning in social, occupational, and psychological domains on a scale of 0 to 100, with lower scores indicating higher impairment | Depression severity | Being hospitalised due to depression (SCAN) or exhibiting high levels of impairment during two worst depressive episodes (score of 30 or less). These two variables were combined and dichotomised into being hospitalised due to depression or scoring 30 or less on GAF | Prospectively | Binary  (1 – yes, 0 – no) | T1 (mean age 12) |
| **The Hospital Anxiety and Depression Scale (HADS)**  (Zigmond & Snaith, 1983) | A self-assessment scale was developed to detect depression and anxiety symptoms. Anxiety subscale used | Anxiety symptoms | Parents with a total score of 11 or more – a validated cut-off score for clinically relevant anxiety – were classified as experiencing anxiety (Zigmond & Snaith, 1983; Sellers et al., 2013) | Prospectively | Binary  (1 – yes, 0 - no) | T1 (mean age 12) |
| **Adult Self-Report Questionnaire** **(ARS)**  (Achenbach & Rescorla, 2003) | A 24-item self-report questionnaire assessing antisocial behaviour. One item, ‘I have never been arrested,’ was deleted since it referred to lifetime behaviour | Antisocial behaviour symptoms | A total antisocial behaviour score was dichotomised using a cut-off of 1 standard deviation above the sample mean (≥13) (Sellers et al., 2013) | Prospectively | Binary  (1 – yes, 0 - no) | T1 (mean age 12) |
| **Alcohol Use Disorders Identification Test (AUDIT)**  (Saunders et al., 1993) | A 10-item questionnaire assessing harmful drinking within the past year | Harmful drinking | Parent was considered to exhibit harmful drinking if they scored 13 or greater on the screening tool (Saunders et al., 1993; Sellers et al., 2013) | Prospectively | Binary  (1 – yes, 0 - no) | T1 (mean age 12) |
| **-** | - | Comorbid MH conditions | Parents were considered as exhibiting comorbid conditions (i.e., on the binary variable) if they scored above the cut-offs on at least one (i.e., HADS, ARS, AUDIT) screening tool | Prospectively | Binary  (1 – yes, 0 - no) | T1 (mean age 12) |
| **Self-reported questionnaire** | Parents were asked to answer questions about their age, completed education, income, and family composition | Age at child’s birth | Derived by subtracting the offspring age from the parent’s age. A higher score indicates older age at offspring birth | Retrospectively | Continuous | T1 (mean age 12) |
|  |  | Completed university degree | Participating parents were asked about the highest completed education level (i.e., GCSEs, A levels, vocational training, university degree and postgraduate degree). The education variable was derived by combining the latter two and dichotomising into a completed university degree or higher | Retrospectively | Binary  (1 – yes, 0 - no) | T1 (mean age 12) |
|  |  | Employed | The parent was asked about their current employment status (i.e., full-time or part-time employment, unemployment, full or part-time education). Employment variable was derived by combining and dichotomising variables into currently in full- or part-time employment | Prospectively | Binary  (1 – yes, 0 - no) | T1 (mean age 12) |
|  |  | Single parent | The parent was asked if they have a partner | Prospectively | Binary  (1 – yes, 0 - no) | T1 (mean age 12) |
|  |  | Family income below £ 20,000 | Parent was asked to select the category that best reflected their gross family income (i.e., up to £10 000, £10,000 – £20,000 etc.). The ordinal variable was dichotomised into gross annual family income below £ 20,000 | Prospectively | Binary  (1 – yes, 0 - no) | T1 (mean age 12) |
|  | **Offspring characteristics** | | | | |  |
| **The Wechsler Intelligence Scale for Children Fourth Edition (WISC-IV)**  (Wechsler, 2003) | A scale that measures intellectual performance as a multidimensional construct | IQ | A full-scale IQ score was used, with a higher score indicating higher intellectual performance. | Prospectively | Continuous | T1 (mean age 12) |
| **Self-reported questionnaire** | Offspring were asked to answer questions about their sex, age, education, living situation, and employment status | Sex | Biological sex | Prospectively | Binary  (1 – female, 0 – male) | T1 (mean age 12) |
|  |  | Age | Age at last birthday | Prospectively | Continuous | T1 (mean age 12) |
|  |  | Lives with parents | Offspring were asked where they currently live (i.e., parental home, residence halls, owned or rented accommodation, etc.). The categorical variable was dichotomised into lives in a parental home | Prospectively | Binary  (1 – yes, 0 – no) | T4 (mean age 23) |
|  |  | Has a university degree or studying | Offspring were asked about the highest completed education level (i.e., GCSEs, A levels, vocational training, university degree and postgraduate degree). They were also asked if they are currently in full or part-time education. These two variables were combined and dichotomised into a university degree or currently in part or full-time education | Prospectively | Binary  (1 – yes, 0 -no) | T4 (mean age 23) |
|  |  | Employed | The variable was dichotomised into currently in full or part-time employment | Prospectively | Binary  (1 – yes, 0 – no) | T4 (mean age 23) |
|  |  | Married or has a partner | Offspring were asked if they were married or had a live-in partner. These two variables were combined and dichotomised into married or had a partner | Prospectively | Binary  (1 – yes, 0 – no) | T4 (mean age 23) |
|  | **Protective factors** | | | | |  |
| **The Iowa Family Interaction Rating Scales (IFIRS)**    (Melby et al, 1993) | It is a global observational coding system developed to assess individual, dyadic, and group-level interactions between family members. A 6-item warmth subscale was used | Parent warmth | Parent self-reported warmth towards their offspring. All items were reverse coded, and the total score was used, indicating higher parental warmth toward their offspring | Prospectively | Continuous | T1 (mean age 12) |
|  |  | Sibling warmth | Sibling self-reported warmth towards the study child. All items were reverse coded, and the total score was used, indicating higher parental warmth toward their offspring | Prospectively | Continuous | T1 (mean age 12) |
| **Perceived Social Support Scale (PSSS)**  (Kessler et al., 1994) | A 3-item scale assessing the level at which respondents believe different individuals (i.e., parents, spouses, children, co-twins, other relatives, and friends) would listen, understand them, and be willing to help. | Co-parent support | The total score of co-parent’s support was derived by summing up the three items, with a higher score indicating more social support from a parent | Prospectively | Continuous | T1 (mean age 12)  T4 (mean age 23) |
| **The Five Minute Speech Sample (FMSS)**  (Caspi et al., 2004) | A modified version of FMSS was used to assess parents’ warmth and hostility. Parents were asked to describe their child by using prompt questions. Two additional prompts (i.e., “What are the strengths or good points?” and “What is it like being their mum or dad? What are the good things?”) were added to elicit responses relevant to resilience | Parent positive expressed emotion (EE) | Trained researchers coded positive expressed emotions of index parents about adolescents according to tone and content of speech samples (range 0-5), with a higher score indicating more positive expressed emotion towards the child (Collishaw et al., 2016) | Prospectively | Continuous | T1 (mean age 12)  T4 (mean age 23) |
| **Strengths and Difficulties Questionnaire (SDQ)**  (Goodman et al., 2000) | A 25-item behavioural questionnaire for children and adolescents. A 5-item peer problem scale was used | Peer relationship quality | The total score of the peer relationship subscale was used to assess various aspects of peer relationships. Items were reverse coded so a higher score would reflect more positive relationships | Prospectively | Continuous | T1 (mean age 12)  T4 (mean age 23) |
| **Peer relationship quality** **questionnaire (PRQ)**  (Rawal et al., 2013) | A ten-item questionnaire devised for the study that assesses adolescents' social esteem and peer inclusion (i.e., “Children in my class are friendly to me”) | Perceived friendships | The total score was used so that the higher score indicates better relationships | Prospectively | Continuous | T1 (mean age 12) |
| **Your health, habits and beliefs questionnaire** | A self-reported questionnaire was devised for the study that assesses lifestyle factors such as fruit intake, exercise frequency, and extracurricular activities. | Out-of-school activities | Parent-reported item "Attendance at clubs or other organised out-of-school activities (at least monthly)" | Prospectively | Binary  (1 – yes, 0 – no) | T1 (mean age 12)  T4 (mean age 23) |
|  |  | Physical exercise | Frequency of exercise was derived by combining two self-reported items (“How often do you exercise (intense enough to be out of breath)?” and “How often do you play sport?”) into a single dichotomised variable (“Intense exercise or sport more than once a week”). | Prospectively | Binary  (1 – yes, 0 – no) | T1 (mean age 12)  T4 (mean age 23) |
| **The Generalized Self-Efficacy Scale (GSE)**  (Jerusalem & Schwarzer, 1995) | Self-reported scale assessing individual’s ability to overcome problems, achieve difficult tasks and cope with adversities (i.e., “If I am in trouble, I can usually think of a solution”) | Self-efficacy | The total score was used so that the higher score indicates better relationships | Prospectively | Continuous | T2 (mean age 14) |
| **The Affective Go/ No Go Task (AGN)**  (Murphy et al., 1999) | The digital and automated cognitive task that assesses affective biases in attention set-shifting and deficits in inhibitory control while processing emotional information | Inhibitory control | During the task, participants were presented with the target valence (i.e., positive or negative word) and asked to press the key when the word matched the target valence and withhold a response to non-target words (distractors). The total number of commission errors (incorrect responses) in all trials was used, and higher errors indicated worse inhibitory control | Prospectively | Continuous | T3 (mean age 15) |
| **Cambridge Gambling Task (CGT)**  (Clark et al., 2008) | The digital and automated cognitive task that assesses risk-taking, decision making and time, delay aversion, and impulsivity | Risk adjustment | During the task, the participant was presented with the row of 10 boxes, with some blue and some red (i.e., the ratio varied between the stages). The yellow token was hidden in one of the boxes, and the participant had to decide if it was hidden in red or blue. Every participant started with 100 points and tried to maximise them by betting a selected portion of their points on the chosen colour under which, in their opinion, the token was hidden. The location where the token was hidden is revealed, and the bet amount is added or subtracted from the total number of points based on the correctness of the decision made. The task consisted of 8 blocks of 9 trials | Prospectively | Continuous | T3 (mean age 15) |
| **The Dysfunctional Attitudes Scale for Children (DAS-C)**  (D'Alessandro & Burton, 2006) | It is a 22 items self-reported scale developed to assess the presence and intensity of dysfunctional attitudes (i.e., “ I can be happy only if everybody I know likes me”, “Young people have to be the best at everything they do”) | Dysfunctional attitudes | An electronic scale version recorded agreement with each item as in the paper version and response time latency in milliseconds. The total score was used, with higher scores indicating more dysfunctional attitudes | Prospectively | Continuous | T3 (mean age 15) |
|  | **Mental health outcomes** | | | | |  |
| **The Child and Adolescent Psychiatric Assessment (CAPA)/ The Young Adult Psychiatric Assessment (YAPA)**  (Angold et al., 1999; Angold & Costello, 2000) | It is a validated interviewer-based semi-structured diagnostic interview used to assess psychiatric disorders and symptoms over the preceding three months. CAPA was used during the first 3 assessments, while YAPA was used in the fourth. For the first 3 assessments, offspring and parent self-reported symptoms were combined using the highest rating per item at the interview level, while only offspring-reported symptoms were used in the fourth assessment | No DSM-IV diagnosis | A binary variable has been derived to capture individuals who did not meet the diagnostic criteria for any of the depressive, anxiety, eating, and behavioural disorders | Prospectively | Binary  (1 – yes, 0 – no) | T1 (mean age 12)  T2 (mean age 14)  T3 (mean age 15)  T4 (mean age 23) |
|  |  | No minor depression | Minor depression was defined a low mood or irritability that lasted for at least 2 weeks and at least one other MDD symptom. Individuals meeting at least one of the criteria were considered as meeting criteria for minor depression. | Prospectively | Binary  (1 – yes, 0 – no) | T1 (mean age 12)  T2 (mean age 14)  T3 (mean age 15)  T4 (mean age 23) |
|  |  | No suicidal behaviour | Suicide-related behaviour was assessed by combining two binary items (i.e., ‘Suicidal behaviour (attempt)’ and ‘Suicidal thoughts’) from CAPA or YAPA and one item (i.e., ‘I thought about killing myself”) from the Moods and Feelings Questionnaire (MFQ) (Angold & Costello, 1988). Individuals answering affirmatively to at least one of these items were considered as manifesting suicide-related behaviours | Prospectively | Binary  (1 – yes, 0 – no) | T1 (mean age 12)  T2 (mean age 14)  T3 (mean age 15)  T4 (mean age 23) |
|  |  | Depressive symptoms | Symptom counts were derived based on the interview responses, with larger scores indicating more symptoms experienced. For categorical mental health outcomes, depressive and behavioural symptoms were dichotomised based on the median (3 symptoms) and used as a binary variable. For better-than-expected mental health outcomes (residual scores), continuous variables of depressive, behavioural, and anxiety symptom counts were used. | Prospectively | Binary (1 – 3 or more symptoms; 0 – < 3 symptoms) and continuous | T1 (mean age 12)  T2 (mean age 14)  T3 (mean age 15)  T4 (mean age 23) |
|  |  | Behavioural symptoms |  | Prospectively | Binary (1 – 3 or more symptoms; 0 – < 3 symptoms) and continuous | T1 (mean age 12)  T2 (mean age 14)  T3 (mean age 15)  T4 (mean age 23) |
|  |  | Anxiety symptoms |  | Prospectively | Continuous | T1 (mean age 12)  T2 (mean age 14)  T3 (mean age 15)  T4 (mean age 23) |

**Appendix S3. Missing data considerations and multiple imputation**

To account for selective attrition and address missing data, multiple imputation by chained equations approach (White, Royston, & Wood, 2011) was used to impute every incomplete variable in the model. First, we investigated the missing patterns in the data and determined the distribution of missing variables, the percentage of missingness, and other diagnostics that would help to build an imputation model (see **Table S2**). Then, to determine if missing values are missing at random (MAR), we identified predictors of non-attendance by comparing individuals that had data on mental health across all four assessments (n = 125), those that had missing data on categorical mental health outcome but participated at the fourth assessment (n = 63), and those that did not participate at the 4^th^ assessment (n = 145). Four individuals unexposed to parental depression were excluded, resulting in a total sample of 333 participants (for more details, see **Figure S2**). These three groups were compared on baseline risk exposures, parent and offspring demographic characteristics, and mental health variables. As demonstrated in **Table S3**, compared to the other two groups, individuals who did not participate in the fourth assessment were more likely to have parents with less severe parental depression characteristics (i.e., their parents were less likely to experience antenatal depression) and were more likely to be from lower socio-economic status families – their parents were younger at birth, a larger percentage had a family income below ￡20 000, and a smaller percentage of their parents had a university degree or were employed at baseline assessment. Furthermore, the IQ of non-participating offspring was lower than that of participants in the other two groups, and a larger percentage experienced mental health problems at baseline –diagnosed with DSM disorder or experienced 3+ depressive symptoms.

We started developing an imputation model by specifying the missing and complete variable types and building an imputation model using only variables included in the analyses. Then, another model was built using one auxiliary variable for each variable of interest. In our case, the auxiliary variable was usually a variable related to non-participation or the same variable assessed at a different time point (time point 2 or 3) with other missing data patterns and strong associations (but not too strong) with the variable of interest. The *dryrun* function was used to run an imputation model and identify potential problems, such as perfect predictions, without actually generating 100 datasets. Perfect prediction and other imputation problems were dealt with by excluding auxiliary variables causing the problem or, if necessary, using the *augment* function that performs augmented regression by adding a few observations with small weights that avoids perfect prediction. Two separate imputation models were built, one for the categorical mental health outcome groups and one for the analyses using residual scores. All core analysis variables (i.e., risk exposures, confounders, and outcomes) were included in the imputation equation for every imputed variable. In addition, we used tailored imputation equations to include other variables when they were relevant (e.g., all family protective factors were used to impute one another but were not included to impute variables from other categories, such as parental depression or demographic characteristics). Tailored imputation equations were used to ensure that variables with missing data were imputed using all necessary variables (e.g., those from the same analysis model) and additional useful variables (e.g., those highly correlated). For the categorical mental health outcome imputation model, binary mental health variables used to derive categorical mental health outcomes across the study period were imputed separately and then combined to passively impute the categorical variable, which was included in the imputation equations for all analysis variables in categorical multiple imputation model. This was done to impute the categorical variable as accurately as possible and also prevent bias from excluding the exact outcome used in the analysis from the imputation model. Predictive mean matching was used for variables with a skewed distribution. We used ten cycles of regression-switching and generated 100 imputed datasets. Convergence plots were used for model diagnostics, and Monte Carlo errors were examined to ensure that 100 imputed datasets were sufficient (White et al., 2011). The code for exploring missing data patterns, multiple imputation models, and analyses with imputed data is available on Github: <https://github.com/padaigaitee/EPAD-mental-health-resilience>.

***Table S2.*** *Characteristics of imputed variables.*

| **Category** | **Variable** | **Time** | **N and % of missing (within total; N = 337)** | **N and % of missing (within T4; N = 188)** | **Type of variable** | **Distribution** |
| --- | --- | --- | --- | --- | --- | --- |
| **Demographics (parent)** | Parent’s (index) age at child’s birth | T1 | - | - | Continuous | - |
|  | Mothers (index) | T1 | - | - | Binary |  |
|  | Completed university degree | T1 | 23 (6.9%) | 7 (3.7%) | Binary |  |
|  | Employed | T1 | 24 (7.2%) | 7 (3.7%) | Binary |  |
|  | Single parent | T1 | 35 (10.5%) | 10 (5.3%) | Binary |  |
|  | Family income below ￡20 000 | T1 | 41 (12.3%) | 12 (6.4%) | Binary |  |
| **Demographics (offspring)** | IQ | T1 | 19 (5.7%) | 14 (7.5%) | Continuous | Normal |
|  | Lives with parent | T4 | 190 (57%) | 45 (23.9%) | Binary |  |
|  | Completed university degree or studying | T4 | 192 (57.7%) | 47 (25%) | Binary |  |
|  | Employed | T4 | 195 (58.6%) | 50 (26.6%) | Binary |  |
|  | Married or lives with a partner | T4 | 190 (57%) | 45 (23.9%) | Binary |  |
| **Risk exposures** | Parental depression age at onset | T1 | 11 (3.3%) | 6 (3.2%) | Continuous | Normal, but negative values were imputed so PMM was used |
|  | Antenatal depression | T1 | 29 (8.7%) | 17 (9%) | Binary |  |
|  | Postnatal depression | T1 | 28 (8.4%) | 17 (9%) | Binary |  |
|  | Family history of depression | T1 | - | - | Continuous | - |
|  | Severe episode (GAF < 30 or hospitalisation) | T1 | 6 (1.8%) | 5 (2.7%) | Binary |  |
|  | Comorbid conditions | T1 | 25 (7.5%) | 9 (4.8%) | Binary |  |
| **Predictors (protective factors)** | Parent depression remission | T1-T3 | 54 (16.2%) | 10 (5.3%) | Binary |  |
|  | Parent warmth | T1 | 24 (7.2%) | 7 (3.7%) | Continuous | Left skew |
|  | Parent positive EE | T1 | 53 (15.9%) | 27 (14.4%) | Continuous | Left skew |
|  | Co-parent support | T1 | 5 (1.5%) | 3 (1.6%) | Continuous | Flat |
|  | Sibling warmth | T1 | 73 (21.9%) | 40 (21.3%) | Continuous | Normal but negative values were imputed, so PMM was used |
|  | Parent-reported peer relationship quality | T1 | 20 (6%) | 6 (3.2%) | Continuous | Left skew |
|  | Adolescent-reported peer relationship quality | T1 | 23 (6.9%) | 5 (2.7%) | Continuous | Left skew |
|  | Adolescent perceived friendships | T1 | 29 (8.7%) | 8 (4.3%) | Continuous | Left skew |
|  | Self-efficacy | T2 | 83 (24.9%) | 30 (16%) | Continuous | Normal |
|  | Inhibitory control | T3 | 80 (24%) | 21 (11.2%) | Continuous | Right skew |
|  | Risk adjustment | T3 | 80 (24%) | 20 (10.6%) | Continuous | Normal |
|  | Dysfunctional attitudes | T3 | 71 (21.3%) | 13 (6.9%) | Continuous | Right skew |
|  | Out-of-school activities (monthly) | T1 | 32 (9.6%) | 11 (5.9%) | Binary |  |
|  | Intense physical exercise (> once a week) | T1 | 25 (7.5%) | 7 (3.7%) | Binary |  |
| **Confounders** | Offspring’s sex | T1 | - | - | Binary |  |
|  | Offspring’s age | T1 | - | - | Continuous | - |
|  | Completed university degree (mother) | T1 | 23 (6.9%) | 7 (3.7%) | Binary |  |
| **Outcomes** | Any DSM disorder | T1 | - | - | Binary |  |
|  | 3 or more depressive symptoms | T1 | 6 (1.8%) | 2 (1.1%) | Binary |  |
|  | 3 or more behavioural symptoms | T1 | 17 (5.1%) | 15 (8%) | Binary |  |
|  | Suicidal ideation | T1 | 4 (1.2%) | 3 (1.6%) | Binary |  |
|  | Minor depression | T1 | - | - | Binary |  |
|  | Any DSM disorder | T2 | 49 (14.7%) | 11 (5.9%) | Binary |  |
|  | 3 or more depressive symptoms | T2 | 53 (15.9%) | 15 (8%) | Binary |  |
|  | 3 or more behavioural symptoms | T2 | 52 (15.6%) | 12 (6.4%) | Binary |  |
|  | Suicidal ideation | T2 | 25 (7.5%) | 7 (3.7%) | Binary |  |
|  | Minor depression | T2 | 51 (15.3%) | 13 (6.9%) | Binary |  |
|  | Any DSM disorder | T3 | 53 (15.9%) | 5 (2.7%) | Binary |  |
|  | 3 or more depressive symptoms | T3 | 56 (16.8%) | 6 (3.2%) | Binary |  |
|  | 3 or more behavioural symptoms | T3 | 53 (15.9%) | 5 (2.7%) | Binary |  |
|  | Suicidal ideation | T3 | 31 (9.3%) | 5 (2.7%) | Binary |  |
|  | Minor depression | T3 | 54 (16.2%) | 6 (3.2%) | Binary |  |
|  | Any DSM disorder | T4 | 190 (57.1%) | 45 (23.9%) | Binary |  |
|  | 3 or more depressive symptoms | T4 | 190 (57.1%) | 45 (23.9%) | Binary |  |
|  | 3 or more behavioural symptoms | T4 | 198 (59.5%) | 53 (28.2%) | Binary |  |
|  | Suicidal ideation | T4 | 201 (60.4%) | 56 (29.8%) | Binary |  |
|  | MDD symptom counts | T4 | 190 (57.1%) | 45 (23.9%) | Continuous | Right skew |
|  | CDODD symptom counts | T4 | 198 (59.5%) | 53 (28.2%) | Continuous | Right skew |
|  | GAD symptom counts | T4 | 204 (61.3%) | 59 (31.4%) | Continuous | Flat |
| **Note.** PMM – predictive mean matching; T1 – time point 1; IQ - intelligence quotient; GAF - Global Assessment of Functioning; EE – expressed emotion; DSM - Diagnostic and Statistical Manual of Mental Disorders; MDD – major depressive disorder; CDODD – conduct and oppositional defiant disorder; GAD – generalised anxiety disorder. | | | | | | |

***Table S3.*** *Reasons for non-participation.*

| **Characteristic** | **Complete case**  **(n = 125)** | **W4 attendee with missing data (n = 63)** | **W4 non-attendees (n=145)** | **Statistical test and p-value** |
| --- | --- | --- | --- | --- |
| **M (SD)** | | | | |
| Parental depression age at onset (T1) | 26.46 (8.40) | 26.31 (8.61) | 26.04 (7.90) | F (2, 319) = 0.09, p = 0.914 |
| Number of relatives with a history of depression (T1) | 1.50 (0.70) | 1.49 (0.67) | 1.58 (0.78) | F (2, 330) = 0.49, p = 0.615 |
| Parent’s (index) age at child’s birth (T1) | 29.82 (4.74) | 30.37 (5.90) | 28.05 (5.24) | F (2, 325) = 5.92, p = 0.003 |
| Child IQ (T1) | 100.19 (12.31) | 94.69 (11.74) | 90.31 (12.14) | F (2, 323) = 22.05, p < 0.001 |
| Child age (T1) | 12.34 (2.09) | 12.44 (2.23) | 12.44 (1.87) | F (2, 330) = 0.09, p = 0.912 |
| **N (%)** | | | | |
| Antenatal depression (T1) | 18/ 115 (15.65%) | 3/56 (5.36%) | 9/133 (6.77%) | χ2 (2) = 7.04, p = 0.030 |
| Postnatal depression (up to 1 year after birth; T1) | 58/115 (50.43%) | 20/56 (35.71%) | 55/134 (41.04%) | χ2 (2) = 3.96, p = 0.138 |
| Severe episode (GAF < 30 or hospitalization) (T1) | 38/122 (31.15%) | 16/61 (26.23%) | 37/144 (25.69%) | χ2 (2) = 1.07, p = 0.585 |
| Comorbid conditions (T1) | 48/122 (39.34%) | 28/57 (49.12%) | 63/129 (48.84%) | χ2 (2) = 2.73, p = 0.255 |
| Parent’s sex (female) (T1) | 116/125 (92.80%) | 59/63 (93.65%) | 136/145 (93.79%) | χ2 (2) = 0.116, p = 0.944 |
| Completed university degree (T1) | 43/122 (35.25%) | 23/59 (38.98%) | 17/129 (13.18%) | χ2 (2) = 21.11, p < 0.001 |
| Employed (T1) | 92/122 (75.41%) | 45/59 (76.27%) | 75/128 (58.59%) | χ2 (2) = 10.19, p = 0.006 |
| Single parent (T1) | 13/120 (10.83%) | 7/58 (12.07%) | 20/120 (16.67%) | χ2 (2) = 1.87, p = 0.392 |
| Family income below ￡20 000 (T1) | 28/ 119 (23.53%) | 14/57 (24.56%) | 55/116 (47.41%) | χ2 (2) = 17.50, p < 0.001 |
| Child sex (female) (T1) | 79/125 (63.20%) | 32/63 (50.79%) | 84/145 (57.93%) | χ2 (2) = 2.70, p = 0.259 |
| Any DSM disorder, including ADHD (T1) | 28/125 (22.40%) | 8/63 (12.70%) | 44/145 (30.34%) | χ2 (2) = 7.78, p = 0.020 |
| 3 or more depressive symptoms (T1) | 31/124 (25.00%) | 8/62 (12.90%) | 43/141 (30.50%) | χ2 (2) = 7.10, p = 0.029 |
| 3 or more behavioural symptoms (T1) | 62/121 (51.24%) | 28/52 (53.85%) | 87/143 (60.84%) | χ2 (2) = 2.57, p = 0.277 |
| Suicidal ideation (T1) | 25/124 (20.16%) | 3/61 (4.92%) | 26/144 (18.06%) | χ2 (2) = 7.43, p = 0.024 |
| Minor depression (T1) | 6/125 (4.80%) | 6/63 (9.52%) | 14/145 (9.66%) | χ2 (2) = 2.52, p = 0.284 |

***Appendix S4.* Complete cases results – risk exposures and protective factors assessed in adolescence**

***Table S3.*** *Risk exposures and categorical mental health outcomes.*

|  | **Total sample (N=125)** | **Group** | | | | **Group comparison** |
| --- | --- | --- | --- | --- | --- | --- |
|  |  | **Sustained good MH**  **(n=13, 10.4%)** | **Recovery**  **(n=24, 19.2%)** | **Adult-onset MH problems**  **(n=10, 8%)** | **Chronic MH problems**  **(n=78, 62.4%)** | **Sustained good MH vs all other groups (reference)** |
|  | **M (SD) or N (%)** | | | | | **Unadjusted**  **OR (95% CI)^◊^** |
| **Parental depression characteristics** | | | | | | |
| Parental depression age at onset (T1) | 26.46 (8.40) | 27.33 (10.05) | 25.21 (9.08) | 32.44 (8.20) | 26.03 (7.79) | 1.12 (0.63, 2.00) |
| Number of relatives with a history of depression ( T1) | 1.50 (0.70) | 1.31 (0.48) | 1.67 (1.01) | 1.40 (0.52) | 1.50 (0.64) | 0.67 (0.32, 1.42) |
| Antenatal depression (T1) | 18/115 (15.7%) | 2/12 (16.7%) | 5/22 (22.7%) | 0/9 (0%) | 11/72 (15.3%) | 1.09 (0.22, 5.44) |
| Postnatal depression (up to 1 year after birth) (T1) | 58/115 (50.4%) | 7/12 (58.3%) | 12/22 (54.5%) | 4/9 (44.4%) | 35/72 (48.6%) | 1.43 (0.43, 4.79) |
| Severe episode (GAF < 30 or hospitalization) (T1) | 38/122 (31.1%) | 2/13 (15.4%) | 7/23 (30.4%) | 0/9 (0%) | 29/77 (37.7%) | 0.37 (0.08, 1.75) |
| Comorbid MH conditions (T1) | 48/122 (39.3%) | 1/12 (8.3%) | 8/23 (34.8%) | 2/10 (20%) | 37/77 (48.1%) | 0.12 (0.02, 0.98) |
| **Parent demographic characteristics** | | | | | | |
| Age at child’s birth (T1) | 29.8 (4.74) | 29.38 (5.12) | 29.38 (4.25) | 33.40 (3.86) | 29.8 (4.80) | 0.89 (0.47, 1.69) |
| Completed university degree (T1) | 43/122 (35.2%) | 5/13 (38.5%) | 6/24 (25.0%) | 3/10 (30.0%) | 29/75 (38.7%) | 1.17 (0.36, 3.82) |
| Employed (T1) | 92/122 (75.4%) | 12/13 (92.3%) | 14/23 (60.9%) | 7/10 (70.0%) | 59/76 (77.6%) | 4.35 (0.54, 34.95) |
| Single parent (T1) | 13/120 (10.8%) | 1/12 (8.3%) | 3/23 (13.04%) | 0/10 (0.0%) | 9/75 (12.05%) | 0.73 (0.09, 6.14) |
| Family income below ￡20 000 (T1) | 28/119 (23.5%) | 1/13 (7.7%) | 8/23 (34.8%) | 1/10 (10.0%) | 18/73 (24.7%) | 0.24 (0.03, 1.96) |
| **Offspring demographic characteristics** | | | | | | |
| Child IQ (T1) | 100.19 (12.31) | 106.08 (8.14) | 96.17 (14.67) | 102.30 (7.87) | 100.13 (12.31) | 1.81 (0.95, 3.46) |
| Lives with parents (T4) | 50/122 (41.0%) | 4/13 (30.8%) | 11/24 (45.8%) | 6/10 (60.0%) | 29/75 (38.7%) | 0.61 (0.18, 2.10) |
| Completed university degree or studying (T4) | 71/120 (59.2%) | 10/13 (76.9%) | 13/24 (54.2%) | 10/10 (100.0%) | 38/73 (52.1%) | 2.51 (0.65, 9.66) |
| Employed (T4) | 99/119 (83.2%) | 11/13 (84.6%) | 20/23 (87.0%) | 9/10 (90.0%) | 59/73 (80.8%) | 1.13 (0.23, 5.51) |
| Married or lives with a partner (T4) | 42/122 (34.4%) | 4/13 (30.8%) | 10/24 (41.7%) | 2/10 (20.0%) | 26/75 (34.7%) | 0.83 (0.24, 2.88) |
| **Note.** Continuous predictors were standardised prior to analyses; Sample size ranged from 115 to 125 based on the risk exposure examined; MH – mental health; GAF – Global Assessment of Functioning; T1 – time point 1 (mean offspring age 12); T4 – time point 4 (mean offspring age 23); OR – odds ratio; IQ - intelligence quotient; ^◊^ - LR test used to obtain p-values. | | | | | | |

***Table S4.*** *Risk exposures and better than expected mental health outcomes.*

|  | **Mood-resilience**  **β (95% CI)** | **Behavioural-resilience**  **β (95% CI)** | **Anxiety-resilience**  **β (95% CI)** |
| --- | --- | --- | --- |
| **Parental depression characteristics** | | | |
| Antenatal depression (T1) | -0.25 (-0.78, 0.28) | 0.24 (-0.32, 0.80) | -0.15 (-0.73, 0.42) |
| Postnatal depression (T1) | 0.07 (-0.28, 0.43) | -0.02 (-0.40, 0.35) | 0.04 (-0.34, 0.42) |
| Comorbid MH conditions (T1) | 0.24 (-0.11, 0.59) | 0.38 (0.02, 0.74) | 0.06 (-0.32, 0.43) |
| **Parent demographic characteristics** | | | |
| Age at child’s birth (T1) | 0.01 (-0.18, 0.20) | 0.03 (-0.17, 0.22) | -0.06 (-0.25, 0.13) |
| Completed university degree (T1) | -0.30 (-0.66, 0.06) | -0.10 (-0.47, 0.28) | -0.33 (-0.71, 0.06) |
| Employed (T1) | 0.01 (-0.36, 0.39) | 0.02 (-0.37, 0.41) | 0.06 (-0.34, 0.46) |
| Single parent (T1) | -0.40 (-0.97, 0.17) | -0.11 (-0.75, 0.52) | -0.22 (-0.83, 0.38) |
| Income less than £20 000 (T1) | 0.16 (-0.24, 0.56) | 0.24 (-0.17, 0.66) | 0.03 (-0.39, 0.45) |
| **Offspring demographic characteristics** | | | |
| Child IQ (T1) | -0.10 (-0.29, 0.08) | -0.06 (-0.26, 0.14) | 0.04 (-0.15, 0.23) |
| Lives with parents (T4) | -0.04 (-0.40, 0.31) | 0.15 (-0.21, 0.50) | -0.31 (-0.67, 0.06) |
| Has a university degree or studying (T4) | -0.35 (-0.71, -0.00) | -0.58 (-0.93, -0.22) | -0.23 (-0.59, 0.14) |
| Employed (T4) | -0.34 (-0.77, 0.10) | -0.08 (-0.55, 0.39) | -0.33 (-0.81, 0.14) |
| Married or has a partner (T4) | -0.18 (-0.53, 0.17) | -0.14 (-0.50, 0.23) | 0.06 (-0.31, 0.43) |
| **Note.** βs obtained by manually standardising continuous outcomes and predictors; CI – confidence interval; IQ - an intelligence quotient; T1 – time point 1 (mean offspring age 12); T4 – time point 4 (mean offspring age 23). | | | |

***Table S5.*** *Protective factors for categorical mental health outcome.*

|  | **Total sample**  **(N= 125)** | **Groups** | | | | **Group comparison** | |
| --- | --- | --- | --- | --- | --- | --- | --- |
|  |  | **Sustained good MH**  **(n=13, 10.4%)** | **Recovery**  **(n=24, 19.2%)** | **Late-onset MH problems**  **(n=10, 8%)** | **Chronic MH problems**  **(n=78, 62.4%)** | **Sustained good MH vs all other groups (ref) (n= 125)** | |
|  | **M (SD) or N (%)** | | | | | **Unadjusted**  **OR (95% CI)** | **Adjusted***  **OR (95% CI)** |
| **Family factors** | | | | | | | |
| Parent depression remission (T1-T3) | 85/124 (68.5%) | 13/13 (100%) | 16/24 (66.7%) | 8/10 (80%) | 48/77 (62.3%) | - † | - † |
| Parent warmth (T1) | 36.50 (5.30) | 34.77 (4.15) | 34.75 (6.13) | 39.00 (4.94) | 37.03 (5.08) | 0.67 (0.36, 1.26) | 0.68 (0.35, 1.32) |
| Parent positive EE (T1) | 3.45 (1.00) | 3.83 (0.72) | 2.95 (1.18) | 3.75 (0.71) | 3.49 (0.99) | 1.65 (0.82, 3.33) | 1.64 (0.82, 3.30) |
| Co-parent support (T1) | 2.44 (2.71) | 3.31 (2.81) | 1.96 (2.51) | 5.90 (0.32) | 2.00 (2.60) | 1.43 (0.80, 2.57) | 1.59 (0.86, 2.94) |
| Sibling warmth (T1) | 15.73 (4.50) | 14.70 (4.17) | 15.80 (4.29) | 16.75 (3.85) | 15.74 (4.73) | 0.75 (0.36, 1.57) | 0.72 (0.31, 1.69) |
| **Social factors** | | | | | | | |
| Parent-reported peer relationship quality (T1) | 8.19 (2.04) | 8.00 (2.08) | 8.04 (1.90) | 9.70 (0.67) | 8.08 (2.14) | 0.90 (0.53, 1.56) | 0.89 (0.50, 1.57) |
| Adolescent-reported peer relationship quality (T1) | 8.07 (1.83) | 8.33 (1.30) | 8.50 (1.22) | 8.30 (1.42) | 7.87 (2.08) | 1.18 (0.63, 2.22) | 1.21 (0.63, 2.33) |
| Adolescent perceived friendships (T1) | 19.24 (5.96) | 19.54 (6.62) | 20.96 (4.76) | 21.40 (4.84) | 18.37 (6.23) | 1.06 (0.60, 1.87) | 1.08 (0.61, 1.91) |
| **Cognitive factors** | | | | | | | |
| Self-efficacy (T2) | 28.33 (4.64) | 30.08 (3.66) | 28.04 (6.31) | 29.78 (2.05) | 27.89 (4.35) | 1.62 (0.84, 3.14) | 1.67 (0.81, 3.44) |
| Inhibitory control (T3) | 14.72 (10.85) | 9.42 (6.04) | 16.29 (10.86) | 7.80 (8.12) | 16.12 (11.31) | 0.38 (0.13, 1.12) | 0.35 (0.11, 1.10) |
| Risk adjustment (T3) | 1.20 (0.88) | 1.52 (0.88) | 1.09 (0.89) | 1.46 (1.01) | 1.13 (0.85) | 1.48 (0.86, 2.55) | 1.49 (0.85, 2.61) |
| Dysfunctional attitudes (T3) | 46.53 (15.34) | 40.00 (9.77) | 44.75 (16.42) | 44.60 (11.82) | 48.57 (16.02) | 0.54 (0.25, 1.15) | 0.49 (0.22, 1.09) |
| **Lifestyle factors** | | | | | | | |
| Out-of-school activities (monthly; parent-reported) (T1) | 81/121 (66.9%) | 7/12 (58.3%) | 14/23 (60.9%) | 9/10 (90%) | 51/76 (67.1%) | 0.66 (0.20, 2.23) | 0.63 (0.17, 2.27) |
| Intense physical exercise (> once a week) (T1) | 84/ 124 (67.7%) | 10/13 (76.9%) | 15/24 (62.5%) | 10/10 (100%) | 49/77 (63.6%) | 1.67 (0.43, 6.42) | 1.66 (0.42, 6.54) |
| **Note.** Continuous predictors were standardised prior to analyses; Sample size ranged from 119 to 125 based on the protective factor examined; MH – mental health; T1 – time point 1 (mean offspring age 12); T2 – time point 2 (mean offspring age 14); T3 – time point 3 (mean offspring age 15); EE – expressed emotion; OR – odds ratio; *- analyses adjusted for offspring’s age, sex, and maternal education (completed university degree) at baseline (T1); † - parameter estimate could not be determined due to perfect prediction (nearly 100% prevalence in the group); ◊ - LR test used to obtain p-values. | | | | | | | |

***Table S6.*** *Protective factors for better than expected mental health outcomes.*

|  | **Mood-resilience** | | **Behavioural-resilience** | | **Anxiety-resilience** | |
| --- | --- | --- | --- | --- | --- | --- |
|  | **Unadjusted**  **β (95% CI)** | **Adjusted***  **β (95% CI)** | **Unadjusted**  **β (95% CI)** | **Adjusted***  **β (95% CI)** | **Unadjusted**  **β (95% CI)** | **Adjusted***  **β (95% CI)** |
| **Family factors** | | | | | | |
| Parent depression remission (T1-T3) | -0.16 (-0.53, 0.22) | -0.19 (-0.57, 0.19) | -0.27 (-0.66, 0.11) | -0.28 (-0.67, 0.10) | -0.30 (-0.69, 0.09) | -0.36 (-0.76, 0.03) |
| Parent warmth (T1) | 0.13 (-0.07, 0.33) | 0.13 (-0.08, 0.34) | 0.02 (-0.18, 0.23) | -0.05 (-0.26, 0.16) | 0.18 (-0.04, 0.40) | 0.21 (-0.02, 0.43) |
| Parent positive EE (T1) | -0.08 (-0.28, 0.11) | -0.07 (-0.27, 0.13) | -0.16 (-0.37, 0.05) | -0.16 (-0.37, 0.04) | -0.10 (-0.30, 0.09) | -0.12 (-0.32, 0.09) |
| Co-parent support (T1) | 0.11 (-0.06, 0.28) | 0.06 (-0.13, 0.24) | 0.12 (-0.06, 0.29) | 0.07 (-0.12, 0.26) | 0.00 (-0.18, 0.18) | -0.03 (-0.22, 0.16) |
| Sibling warmth (T1) | 0.10 (-0.11, 0.31) | 0.06 (-0.18, 0.30) | -0.13 (-0.31, 0.04) | -0.09 (-0.29, 0.11) | 0.12 (-0.10, 0.34) | 0.12 (-0.13, 0.37) |
| **Social factors** | | | | | | |
| Parent-reported peer relationship quality (T1) | -0.06 (-0.25, 0.12) | -0.05 (-0.26, 0.15) | -0.05 (-0.25, 0.14) | -0.03 (-0.24, 0.17) | 0.09 (-0.10, 0.28) | 0.05 (-0.16, 0.26) |
| Adolescent-reported peer relationship quality (T1) | -0.18 (-0.33, -0.02) | -0.20 (-0.36, -0.04) | -0.13 (-0.28, 0.02) | -0.10 (-0.26, 0.06) | -0.05 (-0.22, 0.11) | -0.10 (-0.27, 0.07) |
| Adolescent perceived friendships (T1) | -0.11 (-0.27, 0.05) | -0.13 (-0.29, 0.04) | -0.15 (-0.30, 0.00) | -0.13 (-0.29, 0.02) | 0.02 (-0.15, 0.20) | -0.02 (-0.20, 0.16) |
| **Cognitive factors** | | | | | | |
| Self-efficacy (T2) | -0.01 (-0.19, 0.17) | 0.08 (-0.12, 0.27) | -0.04 (-0.21, 0.12) | -0.01 (-0.18, 0.17) | 0.03 (-0.18, 0.24) | 0.08 (-0.14, 0.30) |
| Inhibitory control (T3) | 0.09 (-0.12, 0.30) | 0.04 (-0.18, 0.27) | 0.01 (-0.18, 0.21) | -0.05 (-0.26, 0.16) | -0.11 (-0.34, 0.11) | -0.16 (-0.39, 0.08) |
| Risk adjustment (T3) | -0.15 (-0.31, 0.02) | -0.16 (-0.33, 0.00) | -0.04 (-0.20, 0.11) | -0.04 (-0.20, 0.12) | -0.01 (-0.19, 0.17) | -0.02 (-0.20, 0.17) |
| Dysfunctional attitudes (T3) | 0.17 (-0.01, 0.35) | 0.18 (-0.00, 0.37) | 0.14 (-0.02, 0.30) | 0.18 (0.01, 0.35) | 0.11 (-0.09, 0.30) | 0.12 (-0.08, 0.33) |
| **Lifestyle factors** | | | | | | |
| Out of school activities (monthly; parent-reported) (T1) | 0.07 (-0.29, 0.43) | 0.11 (-0.29, 0.50) | 0.04 (-0.29, 0.37) | -0.05 (-0.40, 0.30) | -0.10 (-0.49, 0.29) | -0.11 (-0.52, 0.30) |
| Intense physical exercise (> once a week) (T1) | -0.13 (-0.48, 0.23) | -0.17 (-0.54, 0.21) | -0.20 (-0.52, 0.12) | -0.33 (-0.65, -0.00) | -0.20 (-0.57, 0.18) | -0.28 (-0.68, 0.11) |
| **Note.** βs obtained by manually standardising continuous outcomes and predictors; *- analyses adjusted for offspring’s age, sex, and maternal education (completed university degree) at baseline (T1); CI – confidence interval; EE – expressed emotions; T1 – time point 1 (mean offspring age 12); T2 – time point 2 (mean offspring age 14); T3 – time point 3 (mean offspring age 15). | | | | | | |

**Appendix S5. Complete cases results – protective factors assessed in young adulthood**

***Table S7.*** *Mental health outcome group differences on protective factors assessed in young adulthood and predictors of sustained good mental health.*

|  | **Total sample**  **(N= 125)** | **Groups** | | | | **Group comparison** | |
| --- | --- | --- | --- | --- | --- | --- | --- |
|  |  | **Sustained good MH**  **(n=13, 10.4%)** | **Recovery**  **(n=24, 19.2%)** | **Adult-onset MH problems**  **(n=10, 8%)** | **Chronic MH problems**  **(n=78, 62.4%)** | **Sustained good MH vs all other groups (ref)** | |
|  | **M (SD) or N (%)** | | | | | **Unadjusted**  **OR (95% CI)** | **Adjusted***  **OR (95% CI)** |
| **Family factors** | | | | | | | |
| Parent positive EE (T4) | 3.79 (1.13) | 3.45 (1.04) | 3.41 (1.14) | 4.10 (0.88) | 3.91 (1.15) | 0.72 (0.39, 1.35) | 0.70 (0.37, 1.34) |
| Maternal social support (T4) | 4.31 (2.40) | 3.85 (2.79) | 4.63 (2.20) | 5.00 (1.83) | 4.20 (2.48) | 0.81 (0.46, 1.42) | 0.80 (0.45, 1.42) |
| **Social factors** | | | | | | | |
| Parent-reported peer relationship quality (T4) | 8.25 (1.96) | 8.73 (1.01) | 8.04 (2.46) | 8.60 (1.65) | 8.20 (1.95) | 1.39 (0.65, 2.98) | 1.37 (0.63, 2.99) |
| Young adult-reported peer relationship quality (T4) | 7.64 (1.68) | 8.15 (0.99) | 8.54 (1.14) | 7.90 (1.73) | 7.18 (1.79) | 1.49 (0.76, 2.92) | 1.50 (0.75, 2.99) |
| **Lifestyle factors** | | | | | | | |
| Social activities (monthly) (T4) | 33/113 (29.2%) | 4/12 (33.3%) | 8/24 (33.3%) | 4/10 (40.0%) | 17/67 (25.4%) | 1.24 (0.35, 4.44) | 1.16 (0.32, 4.20) |
| Intense physical exercise  (> once a week) (T4) | 53/114 (46.5%) | 7/13 (53.8%) | 11/24 (45.8%) | 6/10 (60.0%) | 29/67 (43.3%) | 1.39 (0.44, 4.44) | 1.27 (0.38, 4.23) |
| **Note.** Sample size ranged from 112 to 119 based on the protective factor examined; MH – mental health; EE – expressed emotion; T4 – time point 4 (mean offspring age 23); *- analyses adjusted for offspring’s age, sex, and maternal education (completed university degree) at baseline (T1). | | | | | | | |

|  | **Mood-resilience**  **β (95% CI)** | **Behavioural-resilience**  **β (95% CI)** | **Anxiety-resilience**  **β (95% CI)** |
| --- | --- | --- | --- |
| **Family factors** | | | |
| *Parent positive EE (T4)* | | | |
| Model 1 | -0.01 (-0.20, 0.19) | -0.19 (-0.39, 0.00) | -0.02 (-0.22, 0.18) |
| Model 2 | 0.03 (-0.18, 0.24) | -0.13 (-0.34, 0.07) | -0.01 (-0.22, 0.21) |
| Model 3 | 0.04 (-0.17, 0.24) | -0.13 (-0.34, 0.07) | -0.00 (-0.22, 0.21) |
| *Maternal social support (T4)* | | | |
| Model 1 | 0.02 (-0.05, 0.09) | 0.03 (-0.04, 0.10) | -0.02 (-0.09, 0.06) |
| Model 2 | 0.03 (-0.04, 0.10) | 0.05 (-0.02, 0.12) | -0.02 (-0.10, 0.05) |
| Model 3 | 0.03 (-0.03, 0.10) | 0.06 (-0.02, 0.13) | -0.01 (-0.09, 0.06) |
| **Social factors** | | | |
| *Parent-reported peer relationship quality (T4)* | | | |
| Model 1 | -0.19 (-0.36, -0.02) | -0.16 (-0.35, 0.03) | -0.12 (-0.31, 0.06) |
| Model 2 | -0.15 (-0.34, 0.03) | -0.12 (-0.31, 0.07) | -0.12 (-0.32, 0.07) |
| Model 3 | -0.10 (-0.29, 0.09) | -0.12 (-0.32, 0.08) | -0.09 (-0.30, 0.12) |
| *Young adult-reported peer relationship quality (T4)* | | | |
| Model 1 | -0.40 (-0.57, -0.23) | -0.33 (-0.51, -0.16) | -0.31 (-0.49, -0.13) |
| Model 2 | -0.38 (-0.56, -0.21) | -0.32 (-0.50, -0.14) | -0.34 (-0.53, -0.15) |
| Model 3 | -0.35 (-0.53, -0.17) | -0.33 (-0.51, -0.14) | -0.34 (-0.53, -0.14) |
| **Lifestyle factors** | | | |
| *Social activities (monthly) (T4)* | | | |
| Model 1 | -0.23 (-0.64, 0.18) | 0.01 (-0.43, 0.43) | -0.48 (-0.91, -0.05) |
| Model 2 | -0.14 (-0.56, 0.28) | -0.06 (-0.49, 0.37) | -0.41 (-0.85, 0.03) |
| Model 3 | -0.28 (-0.71, 0.14) | -0.12 (-0.57, 0.33) | -0.51 (-0.97, -0.06) |
| *Intense physical exercise (> once a week) (T4)* | | | |
| Model 1 | -0.11 (-0.48, 0.26) | -0.25 (-0.63, 0.13) | -0.26 (-0.65, 0.12) |
| Model 2 | -0.07 (-0.44, 0.31) | -0.24 (-0.62, 0.15) | -0.24 (-0.63, 0.16) |
| Model 3 | -0.05 (-0.42, 0.32) | -0.23 (-0.61, 0.15) | -0.23 (-0.63, 0.17) |
| **Note.** Sample size ranged from 104 to 123 depending on protective factor examined; EE – expressed emotion; T4 – time point 4 (mean offspring age 23); Model 1 –unadjusted analyses; Model 2 –analyses adjusted for confounders (offspring sex and age, and maternal education (university degree) at baseline); Model 3 – analyses adjusted for confounders and offspring DSM-IV disorder at baseline. | | | |

***Table S8.*** *Young adulthood protective factors associations with better than expected mental health outcomes in young adulthood.*

**Figure S4. Collider bias**


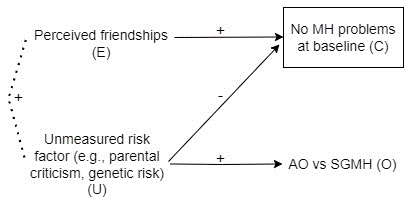


This figure shows a hypothetical Directed Acyclic Graph (DAG) comparing adult-onset (AO) mental health problems and sustained good mental health (SGMH) groups on perceived friendships. E represents exposure/ protective factor (e.g., perceived friendships), C – collider (e.g., not having mental health (MH) problems at baseline), U – unmeasured risk factor(s) (e.g., parental criticism, genetic risk), and O – outcome (e.g., mean or proportion difference between AO and SGMH groups). Arrows in the graph represent hypothesised causal relationships, while + and - symbols represent positive and negative relationships. This hypothetical DAG shows that even when there is no causal association between protective factor (E) and outcome (O) when we condition on collider (C), we induce the association between protective factor (E) and unmeasured risk factor (U), which results in unmeasured risk factor (U) becoming an unmeasured confounder that provides an alternative pathway between protective factor (E) and outcome (O). If an induced association between protective factor (E) and unmeasured risk factor (U), as well as a hypothesised association between an unmeasured risk factor (U) and outcome (O), is positive, this results in detecting a positive association between exposure (E) and outcome (O) (i.e., higher perceived friendships score in AO group compared to SGMH group) even though in reality there is no difference in perceived friendships score between the groups.

**References**

Achenbach, T. M., Rescorla, L. A., McConaughey, S., Pecora, P., Wetherbee, K., & Ruffle, T. (2003). Achenbach system of empirically based assessment. *Handbook of psychological and educational assessment of children: Personality, behavior, and context*, *2*, 406-432.

American Psychiatric Association, A. P., & American Psychiatric Association. (1994). *Diagnostic and statistical manual of mental disorders: DSM-IV* (Vol. 4). Washington, DC: American psychiatric association.

Angold, A., & Costello, E. J. (2000). The Child and Adolescent Psychiatric Assessment (CAPA). *J Am Acad Child Adolesc Psychiatry*, 39(1), 39-48. doi:10.1097/00004583-200001000-00015

Angold, A., Cox, A., Prendergast, M., Rutter, M., Simonoff, E., Costello, E. J., & Ascher, B. H. (1999). *The Young Adult Psychiatric Assessment (YAPA)*. Durham, NC: Duke University Medical Center.

Caspi, A., Moffitt, T. E., Morgan, J., Rutter, M., Taylor, A., Arseneault, L., ... & Polo-Tomas, M. (2004). Maternal expressed emotion predicts children's antisocial behavior problems: using monozygotic-twin differences to identify environmental effects on behavioral development. *Developmental psychology*, *40*(2), 149.

Caspi, A., Moffitt, T. E., Thornton, A., Freedman, D., Amell, J. W., Harrington, H., ... & Silva, P. A. (1996). The life history calendar: a research and clinical assessment method for collecting retrospective event-history data. *International journal of methods in psychiatric research*.

Clark, L., Bechara, A., Damasio, H., Aitken, M. R. F., Sahakian, B. J., & Robbins, T. (2008). Differential effects of insular and ventromedial prefrontal cortex lesions on risky decision-making. *Brain*, *131*(5), 1311-1322.

Collishaw, S., Hammerton, G., Mahedy, L., Sellers, R., Owen, M. J., Craddock, N., ... & Thapar, A. (2016). Mental health resilience in the adolescent offspring of parents with depression: a prospective longitudinal study. *The Lancet Psychiatry*, *3*(1), 49-57.

Costello, E. J., & Angold, A. (1988). Scales to assess child and adolescent depression: checklists, screens, and nets. *Journal of the American Academy of Child & Adolescent Psychiatry*, *27*(6), 726-737.

D’Alessandro, D. U., & Burton, K. D. (2006). Development and validation of the Dysfunctional Attitudes Scale for Children: Tests of Beck’s cognitive diathesis-stress theory of depression, of its causal mediation component, and of developmental effects. *Cognitive Therapy and Research*, *30*, 335-353.

Goodman, R., Ford, T., Simmons, H., Gatward, R., & Meltzer, H. (2000). Using the Strengths and Difficulties Questionnaire (SDQ) to screen for child psychiatric disorders in a community sample. *The British journal of psychiatry*, *177*(6), 534-539.

Kessler, R. C., Kendler, K. S., Heath, A., Neale, M. C., & Eaves, L. J. (1994). Perceived support and adjustment to stress in a general population sample of female twins. *Psychological Medicine*, *24*(2), 317-334.

Melby, J., Conger, R. D., Book, R., Rueter, M., Lucy, L., Repinski, D., ... & Stravos, T. (1993). The Iowa Family Interaction Rating Scales: Iowa Youth and Families Project. 4. *Ames, Iowa: Iowa State University*.

Murphy, F. C., Sahakian, B. J., Rubinsztein, J. S., Michael, A., Rogers, R. D., Robbins, T. W., & Paykel, E. S. (1999). Emotional bias and inhibitory control processes in mania and depression. *Psychological medicine*, *29*(6), 1307-1321.

Powell, V., Agha, S. S., Jones, R. B., Eyre, O., Stephens, A., Weavers, B., . . . Rice, F. (2021). ADHD in adults with recurrent depression. *J Affect Disord, 295*, 1153-1160. doi:10.1016/j.jad.2021.09.010

Rawal, A., Collishaw, S., Thapar, A., & Rice, F. (2013). ‘The risks of playing it safe’: a prospective longitudinal study of response to reward in the adolescent offspring of depressed parents. *Psychological medicine*, *43*(1), 27-38.

Saunders, J. B., Aasland, O. G., Babor, T. F., De la Fuente, J. R., & Grant, M. (1993). Development of the alcohol use disorders identification test (AUDIT): WHO collaborative project on early detection of persons with harmful alcohol consumption‐II. *Addiction*, *88*(6), 791-804.

Schwarzer, R., & Jerusalem, M. (1995). Generalized self-efficacy scale. *J. Weinman, S. Wright, & M. Johnston, Measures in health psychology: A user’s portfolio. Causal and control beliefs*, *35*, 37.

Sellers, R., Collishaw, S., Rice, F., Thapar, A. K., Potter, R., Mars, B., ... & Thapar, A. (2013). Risk of psychopathology in adolescent offspring of mothers with psychopathology and recurrent depression. *The British Journal of Psychiatry*, *202*(2), 108-114.

Wechsler, D. (2012). *Wechsler preschool and primary scale of intelligence—fourth edition.* The Psychological Corporation San Antonio, TX.

White, I. R., Royston, P., & Wood, A. M. (2011). Multiple imputation using chained equations: Issues and guidance for practice. *Stat Med, 30*(4), 377-399. doi:10.1002/sim.4067

Wing, J. K., Babor, T., Brugha, T. S., Burke, J., Cooper, J. E., Giel, R., ... & Sartorius, N. (1990). SCAN: schedules fonr clinical assessment in neuropsychiatry. *Archives of general psychiatry*, *47*(6), 589-593.

Zigmond, A. S., & Snaith, R. P. (1983). The hospital anxiety and depression scale. *Acta psychiatrica scandinavica*, *67*(6), 361-370.
